# Enrichment of the exocytosis protein STX4 in skeletal muscle remediates peripheral insulin resistance and alters mitochondrial dynamics via Drp1

**DOI:** 10.1038/s41467-022-28061-w

**Published:** 2022-01-20

**Authors:** Karla E. Merz, Jinhee Hwang, Chunxue Zhou, Rajakrishnan Veluthakal, Erika M. McCown, Angelica Hamilton, Eunjin Oh, Wenting Dai, Patrick T. Fueger, Lei Jiang, Janice. M. Huss, Debbie C. Thurmond

**Affiliations:** 1 Department of Molecular & Cellular Endocrinology, Arthur Riggs Diabetes and Metabolism Research Institute, City of Hope, Duarte, CA USA; 2 Irell and Manella Graduate School of Biological Sciences, City of Hope, Duarte, CA USA; 3 Comprehensive Metabolic Phenotyping Core, Beckman Research Institute of City of Hope, Duarte, CA USA; 4 Present Address: Amgen, Thousand Oaks, CA USA; 5 Present Address: Washington University School of Medicine, St. Louis, MO USA

**Keywords:** Mechanisms of disease, Type 2 diabetes

## Abstract

Mitochondrial dysfunction is implicated in skeletal muscle insulin resistance. Syntaxin 4 (STX4) levels are reduced in human diabetic skeletal muscle, and global transgenic enrichment of STX4 expression improves insulin sensitivity in mice. Here, we show that transgenic skeletal muscle-specific STX4 enrichment (skmSTX4tg) in mice *reverses* established insulin resistance and improves mitochondrial function in the context of diabetogenic stress. Specifically, skmSTX4tg reversed insulin resistance caused by high-fat diet (HFD) without altering body weight or food consumption. Electron microscopy of wild-type mouse muscle revealed STX4 localisation at or proximal to the mitochondrial membrane. STX4 enrichment prevented HFD-induced mitochondrial fragmentation and dysfunction through a mechanism involving STX4-Drp1 interaction and elevated AMPK-mediated phosphorylation at Drp1 S637, which favors fusion. Our findings challenge the dogma that STX4 acts solely at the plasma membrane, revealing that STX4 localises at/proximal to and regulates the function of mitochondria in muscle. These results establish skeletal muscle STX4 enrichment as a candidate therapeutic strategy to reverse peripheral insulin resistance.

## Abstract (web summary)

STX4 is a regulator of the glucose transporter GLUT4 vesicle fusion at the plasma membrane in skeletal muscle. Here, the authors show that skeletal muscle-specific STX4 enrichment reverses established insulin resistance and improves mitochondrial function in the context of diabetogenic stress.

## Introduction

Diabetes affects over 400 million people worldwide, and this number has quadrupled since 1980 (World Health Organization)^1^. Prediabetes is also on the rise, and half of prediabetic individuals develop type 2 diabetes within 5 years^2^. Prediabetic individuals suffer from elevated blood glucose levels, ranging from 100 to 125 mg/dl, or impaired glucose tolerance. Importantly, prediabetes is reversible; however, current treatments are limited to exercise and dietary modifications. As such, new insulin-sensitising therapeutics are urgently needed^2^. This problem is quickly becoming a crisis, especially as many of the drugs that have been used over the last decade for type 2 diabetes, such as thiazolidinediones, are being withdrawn from the market due to side effects. Therapies that block the progression from prediabetes to type 2 diabetes are especially of interest and a top priority^3^.

In prediabetic and healthy individuals, the skeletal muscle is responsible for over 80% of glucose clearance^4^. Despite the essential role of the skeletal muscle in glucose homeostasis, no directed therapeutics have been developed to restore glucose utilisation specifically in prediabetic skeletal muscle. We previously showed that global upregulation of the exocytosis protein STX4 (gbSTX4tg) causes a twofold increase in glucose uptake, implicating skeletal muscle STX4 in the preservation of glucose homeostasis^5,6^. STX4 mRNA levels are decreased by ~15% in skeletal muscle of diabetic versus healthy mice^7^, suggesting that restoring STX4 expression may improve or *restore* glucose homeostasis. However, whether targeting STX4 only in skeletal muscle is sufficient to improve whole-body glucose utilisation is unknown.

The mechanism linking STX4 enrichment and increased skeletal muscle glucose uptake is thought to involve interaction between STX4 and its cognate Soluble N–ethylmaleimide sensitive factor (NSF) attachment receptor (SNARE) protein partners at the plasma membrane. This interaction facilitates the translocation and fusion of glucose transporter 4 (GLUT4)-bound vesicles with the plasma membrane, thereby increasing insulin-stimulated glucose uptake into the muscle cell^8^. STX4 is the key regulator of GLUT4 vesicle fusion at the plasma membrane in skeletal muscle^5^. Consistent with this, gbSTX4tg increases plasma membrane GLUT4 density in mice^5,6^. However, the repertoire of STX4 functions has recently expanded beyond mediating SNARE exocytosis in other cell types^9^. Hence, it is not known whether STX4-mediated regulation of GLUT4 translocation in skeletal muscle cells is the only mechanism underlying its ability to preserve glucose homeostasis.

Mitochondrial disruption is becoming increasingly recognised as a prominent mechanism underlying progression to diabetes. Under high-fat diet (HFD) conditions, mice exhibit increased mitochondrial fission and mitophagy^10^, with mitochondria appearing vacuolised with unstructured cristae^11^. Mitochondrial fission is controlled by dynamin-related protein (Drp1), which undergoes several post-translational modifications that regulate its function^12^. In particular, phosphorylation of Drp1 at Ser616 (S616) activates mitochondrial fission^13^, whereas phosphorylation of Drp1 at Ser637 (S637) inhibits mitochondrial fission^14,15^. Phosphorylation of Drp1 at S637 by protein kinase A (PKA), or by AMP-activated protein kinase (AMPK)^16,17^, inhibits Drp1 translocation from the cytosol to mitochondria, inhibiting mitochondrial fission. Interestingly, exercise, which can reverse prediabetes, increases pDrp1^S637^ phosphorylation^17^. Consistent with a pro-diabetes role for Drp1-mediated mitochondrial fission, skeletal muscle-specific knockout of calcineurin (an endogenous inhibitor of PKA) yields elongated mitochondria, and confers resistance to HFD-induced obesity^18^. Furthermore, in islet beta cells, AMPK can phosphorylate Drp1 at S637 and prevent changes in mitochondrial morphology in response to diabetogenic stimuli^16^. However, whether STX4 regulates mitochondrial fission is unknown.

The aims of the present study were to evaluate whether transgenic skeletal muscle-specific STX4 enrichment (skmSTX4tg) is sufficient to improve insulin sensitivity and whether the mechanism involves mitochondrial regulation. We demonstrate that skmSTX4tg in mice reverses established insulin resistance, and improves mitochondrial function in response to diabetogenic stress. STX4 associates with Drp1 and mediates Drp1 phosphorylation at S637 in a mechanism involving AMPK in the skeletal muscle. These provocative findings support the notion that skeletal muscle-specific intervention is sufficient to reverse diet-induced insulin resistance, a finding with potential therapeutic implications. They also challenge the view that STX4 acts solely at the plasma membrane and unveil a role for STX4 being on or proximal to the surface of the mitochondria.

## Results

### Skeletal muscle-specific enrichment of STX4 enhances glucose homeostasis in mice

**Fig. 1:**
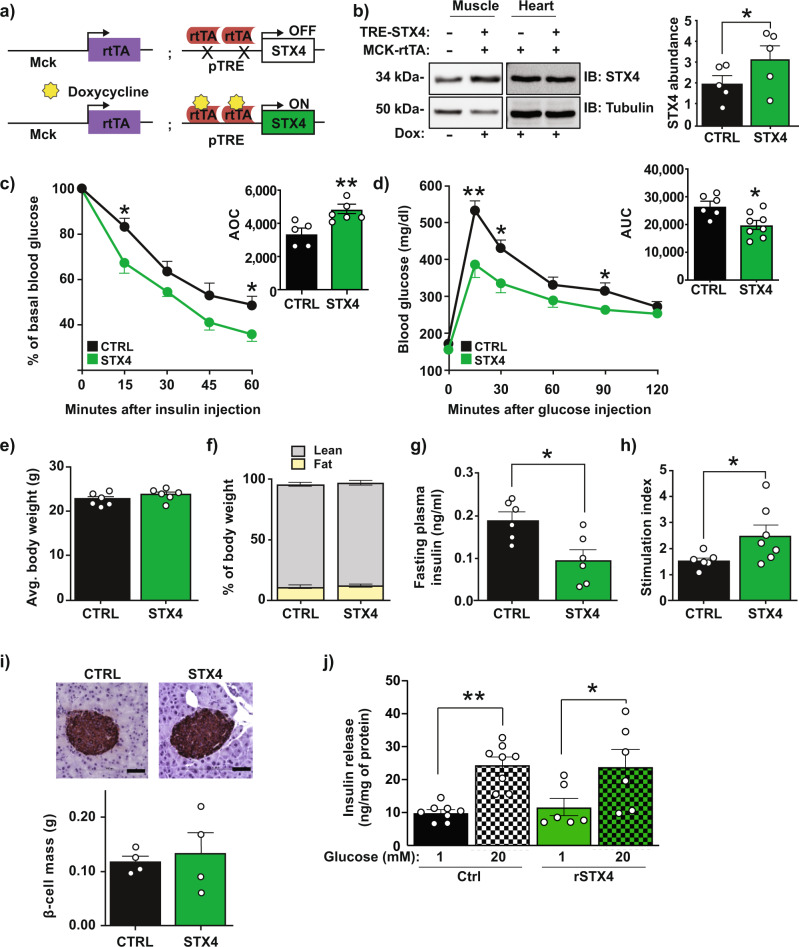
Skeletal muscle-specific STX4 enrichment boosts peripheral insulin sensitivity in female mice. **a** A schematic map of the Tet-On doxycycline (Dox)-inducible model of skeletal muscle-specific STX4 overexpression. **b** Immunoblot of mouse skeletal muscle showing an increase in STX4 expression in Mck-rtTA:TRE-Stx4 mice with 2 mg/ml Dox delivered in drinking water. Heart lysates are shown as a negative control. Quantitation of *n* = 5 mice/group is shown in bar graph to the right. *p = 0.0367 by paired two-tailed t-test. **c** Intraperitoneal insulin tolerance test (IPITT). The bar graph shows data expressed as area over the curve (AOC), [CTRL, *n* = 5; STX4, *n* = 7]. *P* values *15 min=0.0367; *60 min=0.0284; **p = 0.0067 by unpaired two-tailed *t* test. **d** Intraperitoneal glucose tolerance test (IPGTT). The bar graph shows data expressed as area under the curve (AUC), [CTRL, *n* = 6; STX4, *n* = 8]. *P* values **15 min=0.0054; *30 min=0.0180; *90 min=0.0315; *p = 0.0109 by unpaired two-tailed t-test. **e** Body weight (CTRL: Mck-rtTA:TRE-Stx4 without Dox; STX4: Mck-rtTA;TRE-Stx4 with Dox), [CTRL, *n* = 6; STX4, *n* = 6]. **f** Body composition. Grey: lean mass; yellow: fat mass. [CTRL, *n* = 7; STX4, *n* = 8]. **g** Fasted plasma insulin levels in mice fasted 6 h. *p = 0.0109 by unpaired two-tailed t-test. **h** Stimulation index (glucose-stimulated insulin release/basal) from ex vivo pancreatic islets, normalised to % insulin content [CTRL, *n* = 6; STX4, *n* = 6], *p = 0.0272 by unpaired one-tailed t-test. **i** Beta cell mass (*n* = 4/group), scale bar=50 µm. Panels **b**–**i**: data are mean ± SEM (4–6 months old). **j** Insulin release from INS-1 832/13 beta cells treated with conditioned media from skeletal muscle L6 cells overexpressing rat (r)STX4 or vector control (Ctrl) using different passages of L6 and INS-1 832/13 cells (Ctrl, *n* = 8; rSTX4, *n* = 6). Error bars denote mean ± SEM. **p = 0.004; *p = 0.0444 by One-way ANOVA with Tukey’s post hoc test. Source data are provided as a Source Data file.

We generated skmSTX4tg mice on the C57Bl/6 J background, using a Tet-on inducible system to selectively drive *Stx4* expression under the control of a modified muscle creatine kinase (Mck) promoter in response to doxycycline (Dox) in the food or water (Fig. 1a). Dox treatment (2 mg/ml in drinking water) starting at 12 weeks until 25 weeks of age, increased STX4 levels in the skeletal muscle but not the heart (Fig. 1b, *p* = 0.037), as expected^19^. Both insulin sensitivity (intraperitoneal insulin tolerance test, IPITT) and glucose tolerance (intraperitoneal glucose tolerance test, IPGTT) were enhanced in skmSTX4tg female mice (STX4) compared to age-matched (4–6 months old) female non-Dox-induced (CTRL) mice (Fig. 1c, d; increase in IPITT area over the curve [AOC], *p* = 0.007; and decrease in IPGTT area under the curve [AUC], *p* = 0.012). Plasma insulin content sampled during the earliest time point (10 min) of the IPGTT were 0.37 ± 0.11 ng/ml for CTRL and 0.36 ± 0.08 ng/ml for STX4. In male mice, STX4-induced enhancement of IPGTT or IPITT did not reach the significance threshold (Suppl. Fig. 1a, b); enhanced insulin sensitivity in male C57Bl6 mice can be mild by IPITT/IPGTT, requiring clamp analyses to reveal the enhanced insulin sensitivity^5,20^. Single transgenic controls (TRE-STX4 or Mck-rtTA) on Dox, which do not overexpress STX4, showed no change in insulin sensitivity (Suppl. Fig. 1c). SkmSTX4tg did not alter body weight, body composition or organ weights (Fig. 1e, f, Suppl. Table 1). These data recapitulate the phenotypes observed in gbSTX4tg mice^6^, suggesting that STX4 enrichment in the skeletal muscle alone of female mice is sufficient to increase insulin sensitivity and glucose tolerance.

The plasma insulin levels in skmSTX4tg female mice were lower than those in CTRL mice after fasting for 6 hours (Fig. 1g); there was a similar trend at 16 hours, though the magnitude was smaller and the difference did not reach the significance threshold (Suppl. Fig. 1d). We investigated multiple potential explanations for this unexpected effect of a muscle-specific intervention on plasma insulin levels, which are regulated by the pancreatic beta cells. For instance, decreased plasma insulin levels could result from islet cell damage. However, islet function was not decreased—indeed, acute glucose-stimulated insulin secretion (GSIS) ex vivo was enhanced in islets from skmSTX4tg mice relative to CTRL; the islet beta cell mass and islet insulin content were not affected (Fig. 1h, i, Suppl. Fig. 1e). Alternatively, STX4 enrichment-induced insulin sensitivity in the muscle could induce the release of a myokine-type factor that modulates beta cell response, as has been described^21,22^. To test whether STX4 enrichment induces the release of a myokine-like factor, rat skeletal muscle L6 myoblasts were transiently transfected to overexpress STX4 or control DNA (empty vector), and the conditioned media (CM) taken from these skeletal muscle cultures was provided to rat beta cells (INS-1 832/13). No differences in baseline (1 mM glucose) or glucose-stimulated (20 mM glucose) insulin release were detected from beta cells treated with Ctrl- vs. rSTX4-enriched CM (Fig. 1j). Moreover, no differences in the plasma levels of glucagon, cholesterol, triglycerides or adiponectin were evident between skmSTX4tg and CTRL mice fasted for 16 h (Suppl. Fig. 1f–i). The abundances of p^Tyr608^IRS1 and p^Ser473^AKT, which represent early events in the insulin signalling cascade, were also similar between STX4-enriched and Ctrl L6 muscle cells, and between STX4-enriched and CTRL muscle (Suppl. Fig. 1j–l*)*. Although the precise mechanism remains unknown, this phenotypic profile of reduced plasma insulin and enhanced islet function is similar to the phenotype of exercise-trained mice, which also show improved glucose homeostasis^23^.

### STX4 enrichment in skeletal muscle can reverse high-fat-diet-induced insulin resistance

**Fig. 2:**
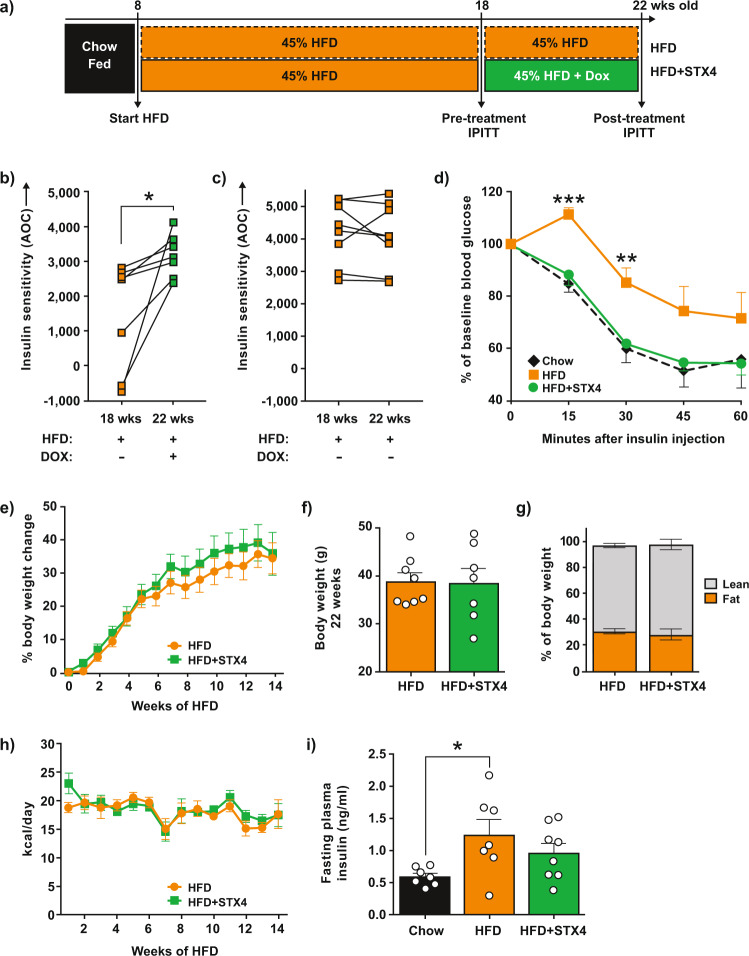
Skeletal muscle-specific STX4 enrichment of high-fat diet (HFD)-fed male mice reverses insulin resistance. **a** Schematic flow: Eight-week-old male Mck-rtTA:TRE-STX4 mice were placed on a 45% HFD for 10 weeks and the IPITT was used to confirm insulin resistance (18 weeks old, pretreatment IPITT). The skeletal muscle-specific STX4 transgene was induced using 625 mg/kg Dox in the HFD in half of the mice (HFD + STX4 group), while the other half remained on only HFD (HFD group). After 4 weeks, the IPITT was performed again (22 weeks old, post-treatment IPITT). **b** IPITT AOC values for HFD + STX4 mice (*n* = 7) pre- and post-treatment. *p = 0.0334 by paired two-tailed t-test. **c** IPITT AOC values for HFD mice (*n* = 8). No significant differences were detected by paired two-tailed t-test. **d** IPITT curves from HFD-fed mice at 18 weeks old (orange; pretreatments IPITT) and the same mice after Dox treatment at 22 weeks old (green; post-treatment IPITT); chow-fed controls (black dashed) provided a reference point. Chow *n* = 5; HFD, HFD + STX4 *n* = 7 each. *P* values ***15 min=0.0004; **30 min= *p* = 0.0013 by unpaired two-tailed t-test comparing HFD and HFD + STX4. **e** Cumulative percent increase in body weight in HFD and HFD + STX4 animals [HFD, *n* = 9; HFD + STX4, *n* = 8]. **f** Body weight at 22 weeks old [HFD, *n* = 8; HFD + STX4, *n* = 7]. **g** Body composition at 22 weeks old after Dox treatment [HFD, *n* = 8; HFD + STX4, *n* = 7]. **h** Average daily calorie consumption per week over the HFD feeding period [HFD, *n* = 9; HFD + STX4, *n* = 8]. **i** Plasma insulin levels in mice at 24 weeks of age, after a 6-h fast [Chow, *n* = 7; HFD, *n* = 7; HFD + STX4, *n* = 8]. Statistics calculated using one-way ANOVA with Tukey’s post hoc test, *p = 0.0298. Error bars in (d-i) represent mean ± SEM. Source data are provided as a Source Data file.

Given that skmSTX4tg was sufficient to improve glucose homeostasis in vivo, we asked whether skmSTX4tg could also remediate HFD-induced insulin resistance. To test this, we developed an insulin resistance rescue paradigm in which a ‘western diet’ model with 45% of kcal from fat was fed to mice for a brief time (10 weeks) to first stage assessment of insulin resistance. The resulting insulin resistant mice were used in the rescue stage to test the capacity of STX4 enrichment to reverse insulin resistance. Previous work has shown that young female mice do not develop significant insulin resistance in this time frame on 60% HFD paradigms^24–28^. Since the basis for the rescue study requires that all groups develop peripheral insulin resistance by the end of the first stage, female mice were not included in the HFD trials. Male mice (8 weeks old) carrying one of both transgenes were fed the HFD and were evaluated for insulin resistance via IPITT at 18 weeks of age (Fig. 2a). Insulin resistance was defined as failure of insulin to lower blood glucose levels by 40% within 60 min in 6-hour-fasted mice. The most insulin resistant mice were provided Dox-containing HFD to induce STX4 expression in the skeletal muscle (HFD + STX4 group), intentionally providing the most stringent challenge for testing the reversal capacity of STX4. The remaining mice were maintained on the HFD without Dox (HFD group). After 4 additional weeks (22 weeks old), the HFD and HFD + STX4 mice were re-assessed for insulin resistance (Fig. 2a). We observed increased insulin sensitivity (increase in AOC for the IPITT) in 100% (7 out of 7) of the HFD-fed skmSTXtg mice after 4 weeks of Dox induction (Fig. 2b, *p* = 0.02), demonstrating that skeletal muscle STX4 can remediate insulin resistance in HFD-fed mice, despite the continued intake of a diabetogenic diet. In contrast, there were no overall trends toward increased insulin sensitivity in the HFD group (*p* = 0.84) over the same 4-week period (Fig. 2c); single transgenic controls also showed no differences in insulin sensitivity (Suppl. Fig. 2a). At 22 weeks, only 4 weeks after initiation of STX4 induction, the insulin sensitivity of the HFD-fed skmSTX4tg mice was indistinguishable from that of age-matched chow-fed mice (Fig. 2d). SkmSTX4tg expression did not impact weight gain, body composition, or caloric intake after HFD feeding (Fig. 2e–h). These results indicate that STX4 enrichment in skeletal muscle can restore insulin sensitivity in a manner independent of adiposity or body weight.

Chronic HFD feeding, in the absence of agents to protect beta cells, causes islet dysfunction^29^. Consistent with this, HFD caused persistent hyperinsulinemia and whole-body glucose intolerance, and skmSTX4tg induction did not remediate these effects (Fig. 2i, *p* = 0.04; Suppl. Fig. 2b, c). While STX4 is known to be protective of beta cells^9^, our data show that the short term 4-week overexpression of STX4 solely in skeletal muscle, initiated after months of palmitate-based HFD exposure, may not be sufficient to elicit significant improvement to beta cell function under diabetogenic conditions, even though skeletal muscle expression of STX4 is coupled to beta cell function under unstressed conditions (Fig. 1h).

We also evaluated markers associated with overnutrition and obesity. We observed HFD-induced hypercholesterolemia, as has been reported^30,31^, in both HFD and HFD + STX4 mice (Suppl. Fig. 2d). Furthermore, HFD caused changes to relative organ weights, as described previously^32^, in both HFD and HFD + STX4 mice (Suppl. Table 2). Plasma levels of glucagon, triglycerides, non-esterified fatty acids (NEFA), and adiponectin were similar among chow-fed, HFD and HFD + STX4 mice fasted for 16 h (Suppl. Fig. 2e–h); hyperleptinemia was observed in both HFD and HFD + STX4 mice (Suppl. Fig. 2i), consistent with overnutrition-associated obesity^33^. In plasma from HFD and HFD + STX4 mice fasted for only 6 h, plasma levels of insulin, glucagon, triglycerides, free glycerol, leptin and adiponectin were also similar (Suppl. Fig. 3a, b, d, e and g, h); however, plasma cholesterol and NEFA were decreased in HFD + STX4 mice relative to HFD mice (Suppl. Fig. 3c and f*)*. Endurance exercise also reduces circulating cholesterol and NEFA, consistent with a model where the benefits of STX4 enrichment and exercise share similar mechanisms^34,35^.

### STX4 enrichment in skeletal muscle can increase spontaneous activity and respiratory exchange ratio

**Fig. 3:**
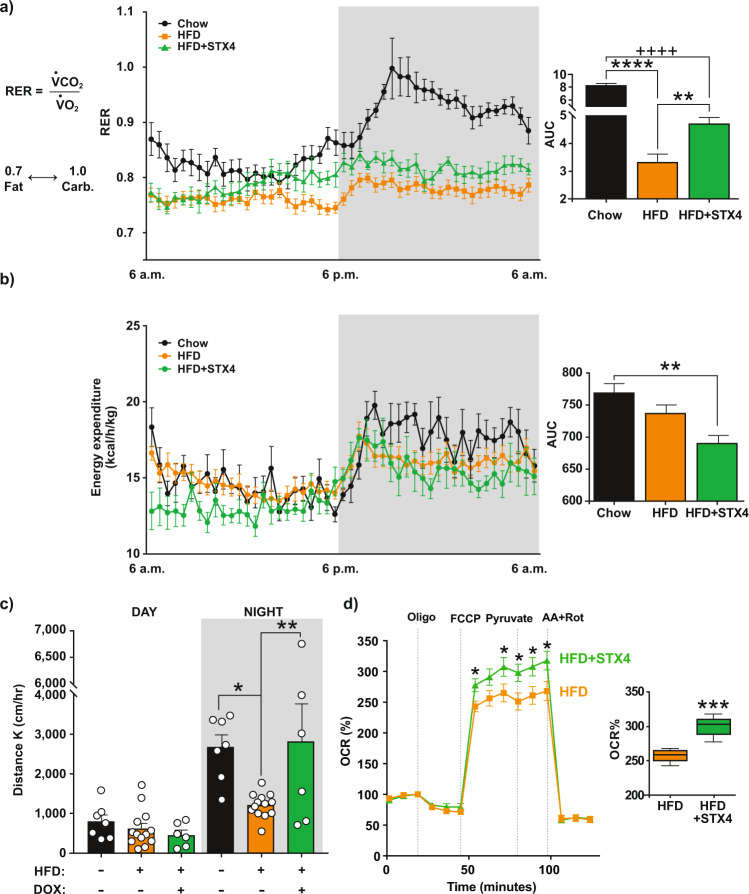
Skeletal muscle-specific STX4 enrichment in HFD-fed mice impacts metabolism and spontaneous physical activity. Metabolic caging analyses of the male mice used in the stages of the feeding paradigm of Fig. 2a; caging data were collected over 24–48 h. **a** Respiratory exchange ratio (RER) time-course over 24 h, with corresponding area under the curve (AUC) calculated as the area under each curve and 0.7 set as the baseline. ****p < 0.0001; ++++*p* < 0.0001; **p = 0.0077 by one-way ANOVA with Tukey’s post hoc test. **b** Energy expenditure time course over 24 h, with corresponding area under the curve calculation. **p = 0.0080 by one-way ANOVA with Tukey’s post hoc test. **c** Average hourly spontaneous activity measured as Distance K during the day (6:00–18:00) and night (18:00–6:00). All data in panels *a-c* are representative of Chow=8, HFD = 16, HFD + STX4 = 6. *p = 0.0120; **p = 0.0083 by one-way ANOVA with Tukey’s post hoc test. *d)* Extracellular flux analysis (Seahorse) to assess the mitochondrial oxygen consumption rate of HFD (*n* = 3, orange) and HFD + STX4 (*n* = 3, green) mice. Inset panel: plot of the change in maximal respiration between the HFD and HFD + STX4 groups. Box plots indicate median (middle line), 25th, 75th percentile (box) and minimum and maximum (whiskers). *p = 0.0158, 0.0493, 0.0217, 0.0291, 0.0256 from left to right, ***p = 0.0001 by unpaired two-tailed t-test. Error bars in (**a**–**d**) represent mean ± SEM. Source data are provided as a Source Data file.

To assess the effects of skmSTX4tg induction on metabolism and behaviour, the male mice from the HFD cohorts were subsequently placed in metabolic caging units for an initial acclimatisation period of at least 24 h, followed by 24 h of parameter measurement. Chow-fed mice had a respiratory exchange ratio (RER) averaging 0.88, indicating a predominantly carbohydrate metabolic fuel source, and HFD-fed mice had an average RER of 0.77, indicating that fat was the predominant metabolic fuel (Fig. 3a), as has been previously described^36^. In contrast to the HFD mice, skmSTX4tg induction in HFD-fed mice significantly shifted the average RER to above 0.81, indicating more carbohydrate use. This skmSTX4tg-induced change in RER was accompanied with decreased energy expenditure in HFD + STX4 mice compared to chow-fed controls (Fig. 3b), suggesting that STX4-enriched mice are more efficient at expending energy (more active, but less energy expenditure). Furthermore, spontaneous physical activity at night (average distance travelled per hour=Distance K) was lower in HFD-fed mice than in chow-fed mice, and the activity levels in HFD + STX4 mice more closely resembled those of the chow-fed mice than the HFD-fed mice (Fig. 3c); Distance K was not correlated with the effects of HFD on body fat or insulin sensitivity (Suppl. Fig. 4a, b, *p* value = 0.36 and 0.30 respectively). Nevertheless, these data suggest that skmSTX4tg induction is sufficient to partially rescue the effects of HFD feeding on metabolism and fully remediate the effects on spontaneous activity.

### STX4 enrichment improves mitochondrial respiration and regulates mitochondrial dynamics

**Fig. 4:**
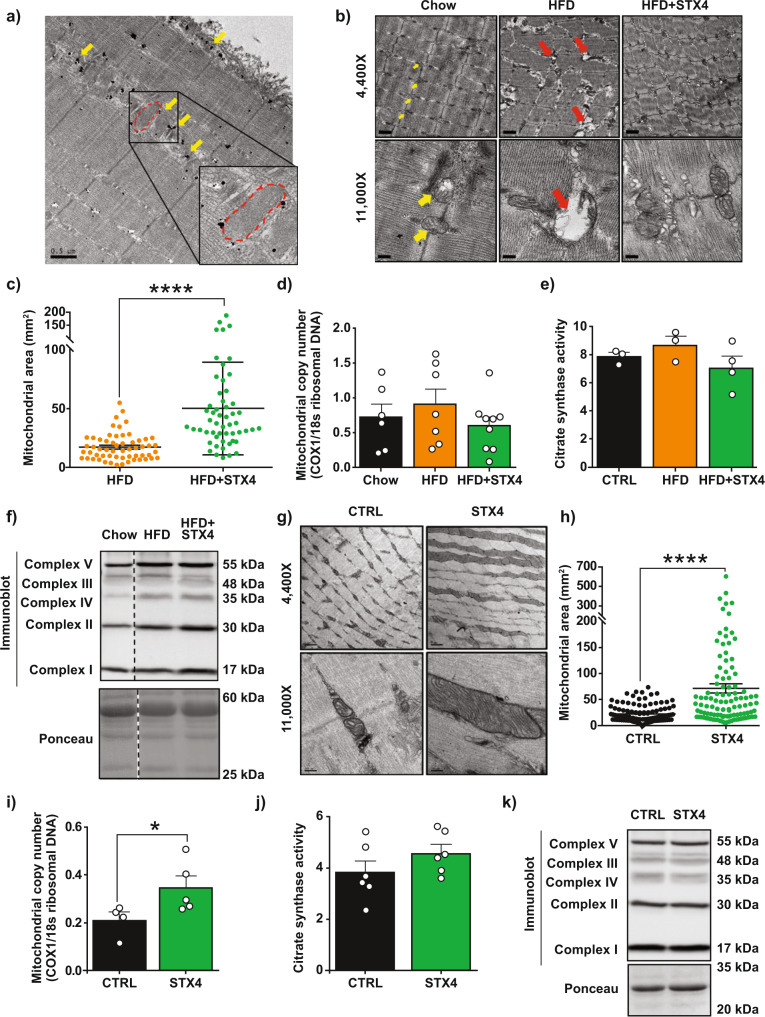
Skeletal muscle-specific STX4 enrichment restores mitochondrial structure. **a** Immunogold STX4 labelling of intramyofibullar (IMF) mitochondria in the tibialis anterior (TA) muscle of STX4-enriched chow-fed male mice. STX4 particles are highlighted with yellow arrows, and a mitochondrion is outlined in red. Scale bar=0.5 µm. **b** Representative TEM images of TA IMF mitochondria at a magnification of 4400X (top) and 11,000X (bottom), in chow-fed, HFD-fed, and HFD + STX4 male mice. Scale bar=1 µm (4400X) and 200 nm (11,000X); mitochondria are indicated with yellow arrows, while red arrows indicate mitochondrial vacuolisation. **c** Mitochondrial area measurements from 4 to 6 skeletal muscle areas, taken at 6500X, from HFD-fed (*n* = 3) and HFD + STX4 (*n* = 4) mice. ****p < 0.0001 by unpaired two-tailed t-test). **d** Mitochondrial DNA (mtDNA) content, measured by comparing the levels of *Cox1* to 18 s ribosomal DNA, in chow-fed (*n* = 6), HFD-fed (*n* = 7) and HFD + STX4 (*n* = 9) male mice. DNA extracted from hindlimb. **e** Citrate synthase activity after a 4.5 min reaction in hindlimb muscle of male chow-fed (*n* = 3), HFD-fed (*n* = 3), and HFD + STX4 mice (*n* = 4). Error bars in (**c**–**e**) represent mean ± SEM. **f** Whole-hindlimb immunoblot of electron transport chain complexes I, II, III, IV and V, in chow-fed, HFD-fed, and HFD + STX4 male mice. Ponceau S was used as loading control. The blot shown is representative of *n* = 3 mice/group. Dashed vertical line indicates the splicing of a lane from within the same gel exposure. **g** Representative TEM images of TA IMF mitochondria at a magnification of 4400X (top) and 11,000X (bottom), in chow-fed CTRL and STX4 female mice. Bar=1 µm (4400X) and 200 nm (11,000X). **h** Mitochondrial area measurements from 4 to 6 skeletal muscle images, taken at 6500X, from CTRL (*n* = 5) and STX4 (*n* = 6) mice. ****p < 0.0001 by unpaired two-tailed t-test). **i** Mitochondrial copy number in chow-fed CTRL (*n* = 4) and STX4 (*n* = 5) female mice. *p = 0.0317 by unpaired two-tailed t-test) and **j** citrate synthase activity in chow-fed CTRL (*n* = 6) and STX4 (*n* = 6) female mice. Error bars in **h**–**j** represent mean ± SEM. **k** Immunoblot of electron transport chain complexes in female chow-fed CTRL and skmSTX4tg mice, representative of *n* = 6 mice/group. Ponceau S is used as a loading control. All TEM images are representative of 3–6 animals/group with 4–6 fields of view evaluated per mouse muscle section, assessed in a blinded fashion by the microscopist. Mitochondrial areas were quantified using Image J. Source data are provided as a Source Data file.

HFD decreases mitochondrial number in human skeletal muscle^37^, changes mitochondrial structure, and reduces oxidative phosphorylation^38,39^. Thus, we assessed whether skmSTX4tg induction alters mitochondrial structure and function. Using the Seahorse XFe24 flux analyser to measure oxygen consumption rates, we determined that primary myofibers from HFD + STX4 mice had greater maximal mitochondrial respiration than myofibers from HFD-fed mice (Fig. 3d). To understand how STX4, a protein characteristically found localised to the plasma membrane, may alter mitochondrial respiratory capacity, we used immunogold labelling with high-resolution TEM to evaluate the subcellular localisation of STX4 in the muscle. STX4 was localised not only to the myofiber sarcolemma but also on or proximal to the surface of the outer mitochondrial membrane (OMM) (Fig. 4a). The specificity of antibody binding was confirmed using a blocking peptide with wild-type muscle and muscle from skmSTX4-KO (knockout) mice, both of which eliminated the immunogold signal at the mitochondrial membranes (Suppl. Fig. 4c). STX4 was also detected in skeletal muscle mitochondrial fractions, further supporting the concept of STX4 being on or proximal to the surface of the mitochondria (Suppl. Fig. 4d).

We next evaluated the structure of the mitochondria in the tibialis anterior (TA) muscle using transmission electron microscopy (TEM). Changes in mitochondrial size and structure are central to complications in obesity and diabetes, and HFD causes an imbalance in mitochondrial fusion-fission, resulting in fragmentation, which increases vacuolisation and reduces cristae uniformity^11^. Muscle mitochondria from the HFD mice appeared fragmented and vacuolised (Fig. 4b, red arrows). By contrast, the mitochondria in the HFD-fed skmSTX4tg muscles appeared very similar to those in chow-fed mice, with little vacuolisation and fragmentation. The mitochondrial area was significantly larger in HFD + STX4 skeletal muscle compared to that in HFD mice (Fig. 4c). We also assessed other measures of mitochondrial function, including mitochondrial DNA copy number and citrate synthase activity. We observed no changes in these parameters among muscles from the chow, HFD and HFD + STX4 groups (Fig. 4d, e). In addition, we looked at potential changes in electron transport chain (ETC) proteins, which could contribute to changes in mitochondrial function and morphology. Again, no changes were observed among the chow, HFD and HFD + STX4 groups (Fig. 4f, Suppl. Figure 4e).

Having established the presence of mitochondrial disruption under diabetogenic conditions, we decided to evaluate the chow-fed skmSTX4tg mice for a similar phenotype. We found that the chow-fed skmSTX4tg mice contained elongated mitochondria with highly organised and structured cristae, compared with chow-fed CTRL mice (Fig. 4g). These findings suggest that skmSTX4tg enrichment increases mitochondrial elongation, while in the context of diabetogenic stress, skmSTX4tg reverses the fragmented phenotype. We observed a population of enlarged mitochondria specifically in skeletal muscle sections from skmSTX4tg mice compared to CTRL mice (Fig. 4h), similar to what was found in HFD + STX4 skeletal muscle (Fig. 4c), suggesting that STX4 may be regulating mitochondrial fission or fusion. The mRNA expression levels for mitochondrial fusion genes were similar for skmSTXtg and CTRL mice (Suppl. Table 3). We did find chow-fed female skmSTX4tg muscle had elevated mtDNA levels (Fig. 4i), most likely due to the enlarged mitochondria, but there were no significant changes across groups in the citrate synthase activity or in ETC subunit levels (Fig. 4j, k, Suppl. Figure 4f). These results were consistent with a model wherein STX4 shifts the balance of mitochondrial fission and fusion toward fusion.

### STX4 overexpression does not impact TCA cycle metabolites in skeletal muscle cells

To assess if STX4 affects mitochondrial metabolism, we looked at tricarboxylic acid (TCA) cycle activity. To do this, we used stable isotope tracing in L6 skeletal muscle myoblasts overexpressing STX4 and compared to empty vector controls. Isotopomer spectral analysis utilises [U-^13^C]glucose to assess the enrichment of TCA cycle intermediates in cells. As the labelled glucose tracer is metabolised, carbons from the glucose are integrated into the downstream metabolites^40^. Isotopic labelling can be measured for various metabolites, such as pyruvate, citrate, and glutamate. We compared the STX4-overexpressing L6 (rSTX4) to Ctrl cells and find that there is no difference in the isotopic labelling between the two groups (Suppl. Figure 5). This suggests that STX4 does not modify glucose metabolism in the cell under basal/ non- insulin-stimulated conditions.

### STX4 overexpression leads to increased Drp1 phosphorylation at S637

**Fig. 5:**
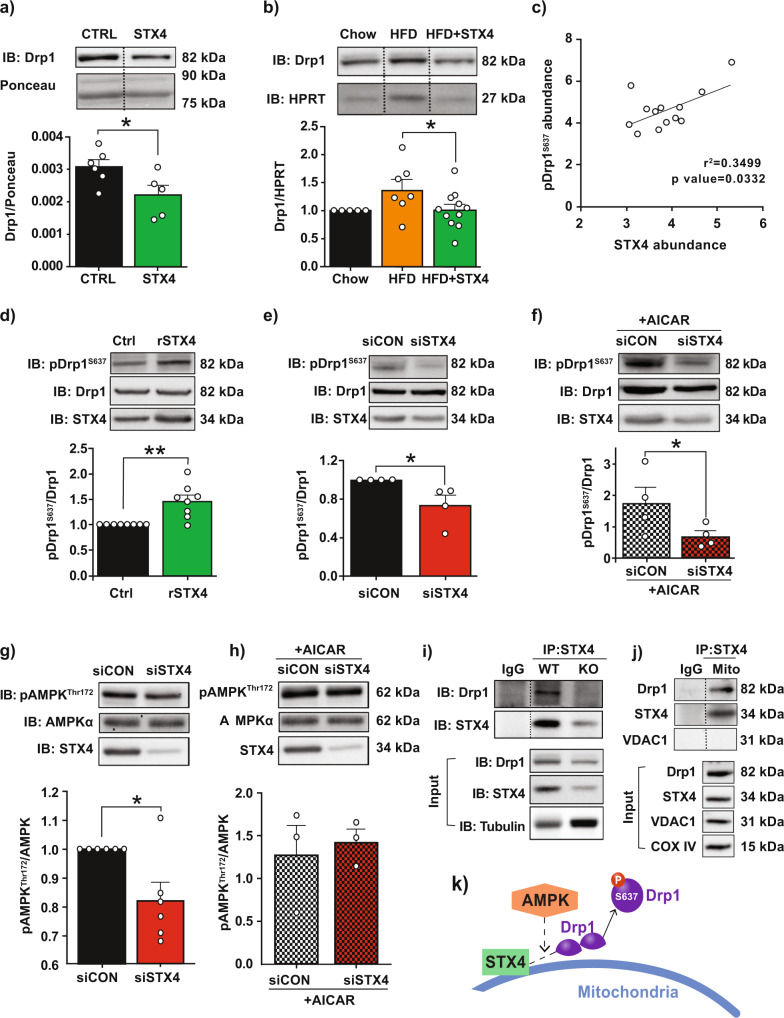
STX4 enrichment leads to increased Drp1 phosphorylation at S637, through a mechanism involving AMPK. **a** Immunoblot of Drp1 levels in chow-fed CTRL or STX4 whole-hindlimb extract. The bar graph shows quantitation of normalised immunoblots, *n* = 5-6/group. Dashed vertical line indicates splicing of a lane from within the same gel exposure. *p = 0.0402 by unpaired two-tailed t-test. **b** Immunoblot of Drp1 levels in chow-fed (*n* = 5), HFD-fed (*n* = 7), and HFD + STX4 (*n* = 11) whole-hindlimb extracts. Each chow served as a control on different gels to control for deviations in blotting/ECL between gels. *p = 0.0374 by one-way ANOVA with Fisher’s LSD test. Error bars in (**a** and **b**) represent mean ± SEM. **c** Scatter plot of pDrp1^S637^ abundance vs. STX4 abundance in skeletal muscle, normalised to HPRT. **d** Skeletal muscle L6 myoblasts overexpressing rat (rSTX4) or vector control (Ctrl) were immunoblotted for pDrp1^S637^ and total Drp1. Data expressed as a ratio of pDrp1^S637^/Drp1 (*n* = 5/group). **p = 0.0013 by unpaired two-tailed t-test. **e** pDrp1^S637^ abundance in L6 skeletal muscle myoblasts expressing STX4 siRNA (siSTX4) or control (siCON). Data expressed as a ratio of pDrp1^S637^/Drp1 (*n* = 7/group). *p = 0.0415 by unpaired two-tailed t-test. **f** Effect of AICAR treatment on pDrp1^S637^ in siCON and siSTX4-treated L6 cells. Data expressed as a ratio of pDrp1^S637^/Drp1 (*n* = 3/group). *p = 0.0492 by unpaired one-tailed t-test). **g** pAMPK^Thr172^ abundance with STX4 knockdown (*n* = 6/group). Data expressed as a ratio of pAMPK^Thr172^/AMPK. *p = 0.0176 by unpaired two-tailed t-test. **h** pAMPK^Thr172^ abundance upon AICAR treatment and STX4 knockdown (*n* = 3/group). Data expressed as a ratio of pAMPK^Thr172^/AMPK. Error bars in (**d**–**h**) represent mean ± SEM. **i** Co-immunoprecipitation of STX4 and Drp1 from skeletal muscle homogenates prepared from WT or skmSTX4-iKO mouse hindlimb muscle; representative of two sets of WT and KO mouse homogenates. **j** Co-immunoprecipitation of STX4 and Drp1 from mitochondrial fractions prepared from WT mouse hindlimb muscle; representative of three coIP experiments each using an independent set of mouse muscle mitochondrial fractions. **k** Schematic illustration of the mechanism of action of STX4, Drp1, and AMPK at the mitochondria in skeletal muscle. Source data are provided as a Source Data file.

The mitochondrial localisation of STX4 and its effects on mitochondrial dynamics led us to assess whether skmSTX4tg regulates the expression of proteins involved in mitochondrial dynamics. We observed that STX4 overexpression decreased the levels of Drp1, a regulator of mitochondrial fission, in hindlimb muscles from mice fed chow or HFD (Fig. 5a, b). Given that Drp1 function is regulated by its phosphorylation state, we assessed Drp1 phosphorylation in the mouse skeletal muscle and found a significant positive correlation between STX4 abundance and Drp1 S637 phosphorylation, which enhances mitochondrial fusion (Fig. 5c); however, there were no significant changes in Drp1 S616 phosphorylation, which enhances fission (Suppl. Fig. 6a). To clarify the mechanism of action, we used L6 skeletal myotubes overexpressing STX4, which show a characteristic robust increase in glucose uptake upon insulin stimulation (Suppl. Fig. 6b, c), mimicking the insulin-sensitising effects of STX4 in skeletal muscle. STX4 overexpression in the L6 cells increases pDrp1^S637^ (Fig. 5d), similar to the effect seen in mouse skeletal muscle (Fig. 5c). To assess if STX4 is required for Drp1 S637 phosphorylation, we transiently transfected L6 myoblasts with non-targeting (siCON) or STX4-targeting siRNA (siSTX4). Compared to controls, siSTX4-treated skeletal muscle myoblasts showed a significant reduction in pDrp1^S637^ (Fig. 5e), which supports the notion that STX4 is required for Drp1 phosphorylation at S637.

To evaluate the extent to which Drp1 modulation alters STX4 metabolic effects, seahorse analyses paired with western analysis of phosphorylation changes to Drp1-S637 and -S616 were performed in an independent set of L6 myoblast studies. We initially demonstrated that STX4 knockdown alone (transient transfection, siRNA) impairs maximal respiration (Suppl. Fig. 6d). These findings complement those from gain-of-function studies in which STX4 overexpression increased maximal mitochondrial OCR in HFD mice (Fig. 3d). STX4 knockdown phenocopies the effects of Drp1 overexpression (via regulation of pDrp1-S637, and not S616) (Suppl. Fig. 6e–i). Further, the decrease in pDrp1-S637 with STX4 knockdown (even with Drp1 overexpression, Suppl. Fig. 6g) provide additional support for the concept that STX4 regulates Drp1. These results complement the other metabolism-based studies, metabolic flux studies using the same cells as well as the metabolic caging studies of the mice, to demonstrate that STX4 modulation regulates mitochondrial function.

### STX4 regulates pDrp1^S637^ in a mechanism involving AMPK but not PKA

PKA has been reported as the primary kinase for Drp1 S637 phosphorylation^41^. To test whether STX4 increases PKA activity to induce pDrp1^S637^, we treated cells with forskolin (activates adenylyl cyclase to elevate cyclic AMP levels), in the presence and absence of STX4 enrichment or knockdown. Changing STX4 expression did not affect PKA activity, either in the presence or absence of forskolin (Suppl. Fig. 7a, b).

AMPK is also reported to phosphorylate Drp1 at S637, preventing HFD-induced changes in the ER and in mitochondrial morphology^16^. We initially confirmed that AICAR, an AMPK activator, could increase the levels of active AMPK (pAMPK^Thr172^) in L6 muscle cells (Suppl. Fig. 7c*)*. Furthermore, AICAR increased pDrp1^S637^ levels (Suppl. Fig. 7d), confirming the ability of AMPK to phosphorylate Drp1 at S637 in the muscle cells. We then tested whether the presence of STX4 is required for AICAR to induce pDrp1^S637^. STX4 knockdown blocked the ability of AICAR to induce pDrp1^S637^ (Fig. 5f), consistent with the concept that AMPK participates in the mechanism by which STX4-mediated Drp1 S637 phosphorylation. In addition, siSTX4 can suppress AMPK activation (pAMPK^Thr172^) (Fig. 5g), but this can be over-ridden by AICAR (Fig. 5h). Together, these data are consistent with a model wherein STX4 induces Drp1 S637 phosphorylation in a mechanism involving AMPK but not PKA.

To further assess the role of STX4 in Drp1 regulation, we immunoprecipitated STX4 to evaluate whether STX4 associates with Drp1. Drp1 was detected in STX4 immunoprecipitates from wild-type skeletal muscle (WT), but not skmSTX4-iKO (KO) muscle, and not in IgG control immunoprecipitates from WT muscle (Fig. 5i). Co-immunoprecipitation reactions performed using isolated WT mouse muscle mitochondrial fractions similarly showed Drp1 detection in STX4 immunoprecipitates, and not in IgG control immunoprecipitates (Fig. 5j). These results support a model wherein STX4 localised on or proximal to the mitochondria associates with Drp1 and, in a mechanism involving AMPK, mediates Drp1 S637 phosphorylation (Fig. 5k).

## Discussion

To the best of our knowledge, this study is the first to show a remediation of peripheral insulin resistance and the reversal of the negative effects of HFD on spontaneous activity, by the selective enrichment of a protein in skeletal muscle. We have shown that STX4, a SNARE protein known for its regulation of GLUT4 translocation and glucose uptake in skeletal muscle, is capable, when enriched, of reversing established HFD-induced insulin resistance. STX4-enriched mice showed a full restoration to normal insulin sensitivity, despite on-going exposure to HFD. This suggests that targeting STX4 might also reverse prediabetes in humans, even in the continued presence of diabetogenic stress. We also provide the unusual finding of STX4 being on or proximal to the mitochondrial surface and mediates mitochondrial dynamics, reverting vacuolised, and fragmented mitochondria in the HFD-fed mice, back to structured, round and organised organelles. Furthermore, STX4 suppresses mitochondrial fission through the phosphorylation of Drp1 in a mechanism involving AMPK.

In this study, we used the most insulin resistant mice to be Dox-induced for STX4 enrichment. While there is documented heterogeneity in the severity of insulin resistance generated by HFD paradigms^42^, the basis for the rescue study requires that all groups develop peripheral insulin resistance by the end of the first stage; this approach is the most stringent way to assess the therapeutic potential of STX4. This approach ensures that STX4 is tested on the animals experiencing the largest effects of diabetogenic stress. We chose a 45% HFD, which is more physiologically similar to the diabetogenic western diet^43^, whereas 60% HFD is not physiologically relevant, and causes irreversible pancreatic beta cell damage.

The current approaches to reverse prediabetes are limited to exercise and dietary modifications. Our findings support that Stx4 enrichment in skeletal muscle may provide an insulin-sensitising therapeutic approach. Although little is known about the links between STX4 and exercise, there are intriguing parallels between the effects of STX4 enrichment and those of endurance exercise on skeletal muscle metabolism. For instance, both interventions improve maximum respiratory capacity and change mitochondrial dynamics. Exercise induces mitochondrial elongation^44^, similar to the effects of STX4. Exercise and STX4 enrichment also both improve glucose tolerance and insulin sensitivity. In addition, exercise-trained mice show increased spontaneous activity levels compared to untrained controls^45^, similar to the increased spontaneous activity of the skmSTX4tg mice. However, if STX4 is truly acting as an exercise mimetic, it is not doing so completely. Mice remain obese after STX4 induction, whereas exercise causes weight loss and reduced percentage of abdominal body fat^45^. This could be due to the fact that the mice are maintained on the HFD, and they display no changes in caloric intake, so the spontaneous physical activity is not reducing their caloric load enough to lead to weight loss.

Our finding that STX4 may localise to the OMM refutes the long-standing concept that STX4 acts only at the plasma membrane^46,47^. Moreover, skmSTX4tg also reduces HFD-induced mitochondrial fragmentation and decreases the abundance of Drp1, a regulator of mitochondrial fission. A hallmark of HFD-induced diabetes is increased mitochondrial fission and mitophagy^48^. It is therefore possible that skmSTX4tg remediates HFD-induced insulin resistance via suppressing mitochondrial fission through Drp1 S637 phosphorylation. Nonetheless, the exact mechanism of how STX4 is mediating mitochondrial fission remains to be determined. In support of this finding, a recent report shows that STX4 can localise to the mitochondrial membrane in a variety of other cell types^49^.

Furthermore, in this study, we have demonstrated that STX4 mediates mitochondrial dynamics by facilitating Drp1 phosphorylation at S637 through a mechanism involving AMPK. STX4-enriched cells showed increased S637 phosphorylation of Drp1. In contrast, knockdown of STX4 decreased pDrp1^S637^, and also decreased AMPK activation. We show here that AMPK activation increases Drp1 S637 phosphorylation in skeletal muscle; this phosphorylation event was previously only shown in beta cells^16^. In addition, we show that STX4 is required for AMPK phosphorylation of Drp1 at S637, as the effects of STX4 knockdown on Drp1 S637 phosphorylation cannot be rescued by AICAR treatment. Lastly, the results of our co-immunoprecipitation experiments suggest that STX4 associates with Drp1 in the mitochondrial fraction of whole-hindlimb mouse muscle. Interestingly we found no effect of STX4 expression on pDrp1^S616^, although it has been reported that mitochondrial fission increases with HFD feeding^10^. However, it is possible that we missed the window to capture pDrp1^S616^ changes, as pDrp1^S616^ is a transient modification and returns to baseline phosphorylation levels within 3 hours of rest after acute exercise^50^.

Although this study provides critical mechanistic insight, we do not know how STX4 regulates AMPK activation and Drp1 S637 phosphorylation. AMPK is controlled by the ratio of AMP to ATP in the cell. The primary mechanism of AMPK activation, especially in metabolic disease conditions, is by preventing AMPK dephosphorylation by AMP, or by low ATP levels (energetic stress). In addition, as a nutrient sensor, AMPK can be activated by cellular glucose levels, and a recent study demonstrates that cellular glucose can activate AMPK regardless of the AMP:ATP ratio^51^. Given that AMPK is a nutrient sensor, there are several ways in which STX4 might regulate AMPK activation: 1) STX4 may modulate the AMP to ATP ratio in skeletal muscle to activate AMPK, or 2) STX4 might activate AMPK independent of AMP/ATP ratios via modulating cellular glucose levels. Given that stable isotope tracing in skeletal muscle cells suggests that STX4 enrichment does not affect glucose metabolites, we propose that STX4 likely alters AMPK activity by regulating AMP/ATP ratios or through an atypical mechanism. We plan to pursue the detailed mechanism in future studies.

In conclusion, we have shown that induction of STX4 selectively in the muscle of HFD-fed, insulin resistant mice restores insulin sensitivity to the level of chow-fed mice. Skeletal muscle-specific overexpression of STX4 phenocopies that of the global STX4 overexpressing mice, pointing to skeletal muscle as the primary driver of whole-body insulin sensitivity. This work supports further investigation of skeletal muscle STX4 enrichment as a therapy to prevent or reverse diabetes. We also found that induction of STX4 in skeletal muscle of HFD-fed mice reduces HFD-induced mitochondrial fragmentation via atypical mechanism that involves STX4 localisation on or proximal to mitochondrial membranes and regulation of Drp1 phosphorylation through a mechanism involving AMPK. However, the precise details of the mechanism are still required to elucidate the full effect of STX4 enrichment in skeletal muscle and determine its potential and validity as a prediabetic therapeutic.

## Methods

### Transgenic mice and feeding studies

All animal experiments were conducted in accordance with the NIH Guide for the Care and Use of Laboratory Animals (National Institutes of Health Publication no. 85-23, revised 1996) and approved by the Institutional Animal Care and Use Committees of City of Hope National Medical Center (Duarte, CA, USA; approval #15023). Single transgenic TRE-STX4 mice were generated on the C57BL6J background as described^19^. Mck-rtTA mice were purchased from the Jackson Laboratory (Bar Harbor, ME, USA) and maintained on the same background. Double transgenic mice were heterozygous for each transgene, obtained from heterozygous matings. Littermates were used as controls. All experiments were repeated with at least 2–3 independent cohorts of mice. Chow-fed mice were housed on Sani-Chip bedding in groups of 3–5 and fed standard maintenance chow (13% of kcal from fat; PicoLab #5053) starting at 4 weeks of age. In the HFD feeding paradigm, 8-week-old skmSTX4tg mice were fed a custom diet (45% of kcal from fat, principally palmitate based; Research Diets #D01030108) *ad libitum* for 10 weeks. The most insulin resistant mice were placed on HFD supplemented with 625 mg Dox (45% of kcal from fat; Research Diets #D17100202) for 4 weeks, whereas the remaining mice fed HFD were maintained on the HFD without Dox. All HFD-fed mice were housed individually on Sani-Chip bedding, 12 h light, 12 h dark cycle; food was measured and changed twice weekly and body weight was measured once a week. STX4 floxed (fl/fl) mice were obtained from Dr. Sidney Whiteheart (University of Kentucky)^52^ and crossed with human skeletal actin (HSA)-rtTA/TRE-Cre recombinase positive mice (JAX # 012433) to produce doxycycline-inducible STX4 fl/fl:Cre+ mice (KO, heterozygous for Cre). Mice were treated with 2 mg/kg doxycycline in water to induce STX4 knockout.

### Glucose and insulin tolerance testing

The IPGTT was conducted in 4–6-month-old mice fasted for 6 hours prior to experimentation (0800–1400 h) and housed individually. The mice were injected (i.p.) with 2 mg/kg D-glucose, which had been diluted in saline and filtered. Blood glucose levels were measured immediately before injection (0 min) and then at 15, 30, 60, 90, and 120 min after injection. Blood (2 µl postinjection) was taken from the mouse tail and diluted with 5 µl saline, then loaded into cuvettes and measured immediately using a HemoCue 120706 Glucose 201 Analyzer. Blood taken before injection was directly loaded into the cuvettes without dilution. For the IPITT, after the blood was collected, the mice were injected intraperitoneally with Humulin R (0.75 units/kg) (Eli Lilly & Co., Indianapolis, IN). Blood taken thereafter was directly loaded into cuvettes without dilution.

### Immunoblot analysis

Mouse whole-hindlimb was extracted and flash-frozen immediately in liquid nitrogen. The hindlimb was then lysed using NP-40 lysis buffer (25 mM HEPES pH 7.4, 1% NP-40, 10% glycerol, 137 M NaCl, 1 mM Na_3_VO_4_, 50 mM NaF, 10 mM NaPP, 10 µg/ml aprotinin, 5 µg/ml leupeptin, 1 µg/ml pepstatin, 1 mM PMSF, and 1 µl of dithiothreitol added per 1 ml of lysis buffer). The samples were rotated at 4^o^C for 5 min and centrifuged at 17,100 × *g* for 5 min. The supernatant was collected and aliquoted into tubes to avoid freeze/thaw protein degradation. The proteins were resolved on hand-casted 10% SDS-PAGE gels, using 20 µg protein/sample/lane, and transferred onto PVDF membrane. Membranes were immunoblotted for STX4 (custom antibody, 1:3000)^53^, Tubulin (Sigma #T5168, 1:5000), Drp1 (Abcam #ab56788, 1:1000), pDrp1^S637^ (Cell Signaling, #4867 S, 1:1000), pDrp1^S616^ (Cell Signaling #3455, 1:1000) AMPK alpha (Cell Signaling #2793, 1:2000) and pAMPKα ^T172^ (40H9) (Cell Signaling # 2535, 1:1000) in TBST + 1% bovine serum albumin (BSA) with sodium azide. For the secondary antibody, Goat Anti-Rabbit IgG (H L)-HRP Conjugate (Bio-Rad #172-1019) or Goat Anti-Mouse IgG (H L)-HRP Conjugate (Bio-Rad #172-1011) (see Suppl. Table 4 for detailed dilutions) was incubated with the blot for 1 h at room temperature (RT). Chemiluminescence was documented using a Bio-Rad ChemiDoc Touch and an enhanced chemiluminescence kit (Amersham ECL Western Blotting detection reagent, GE Healthcare #RPN2106). Images were analysed using Image Lab ver 6.0.1 (Bio-Rad, Hercules, CA).

### Cell culture

L6 GLUT4myc skeletal muscle cells (Kerafast cat# ESK202-FP) where maintained using complete growth media (MEM-α medium supplemented with 10% foetal bovine serum [FBS] and 1% [v/v] antibiotic-antimycotic solution) at 37 ^o^C and 5% oxygen. MEMα medium was purchased from Invitrogen (Carlsbad, CA). In addition, blasticidin (Millipore sigma cat# 203350, 6 µl/ 15 ml media) was added to each passage to select for GLUT4myc cells. Cells were transfected the following day, using K2 transfection reagent: cells were pre-treated with K2 multiplier for 2 h at 10 µl/ml, then DNA (pcDNA3.1 or pcDNA3-STX4) was combined with transfection reagent for 20 min in serum-free media, then the DNA-K2 mixture was added to each well. For siRNA treatment, RNAi Max was used together with OptiMEM according to the manufacturer’s instructions at a concentration of 100 nM and added to each well in a drop-wise fashion. Transfected cells were left overnight and fresh media was added. siSTX4 sequence: 5’-TCACTTTTCTAGCTACCGA-3’ (Ambion). Forskolin (Sigma, cat# F3917) was used at a final concentration of 20 µM and added to the medium for 1 h. AICAR was used at a final concentration of 0.5 mM for 16 h. For insulin stimulation, cells were starved using serum-free media for 50 min, then bovine insulin was added at a final concentration of 100 nM per well. To differentiate the cells, myoblasts were seeded at 10,000 cells/ml into differentiation media (MEM alpha, 2% FBS, and 1% antibiotic-antimycotic), for 7 days to completely differentiate. Upon complete differentiation, the cells were transduced with Ad5-CMV-STX4 or Ad5-CMV control virus for 72 h.

### Morphometric assessment of islet cell mass

Mouse islet morphometry was conducted using pancreatic sections stained with an antibody to insulin. In Brief, pancreata from CTRL or skmSTX4tg mice were fixed with 4% paraformaldehyde, paraffin-embedded, and sectioned longitudinally at 5-μm thickness and 100-μm intervals. The sectioned tissues were deparafinized, rehydrated, blocked in 5% horse serum, and incubated overnight at 4 °C with rabbit anti-insulin antibody^54^. The beta cell area was calculated using the Keyence BZX-Analyzer version 1.3.1.1software. Data shown are representative of three pancreatic sections per mouse. The beta cell mass was calculated by multiplying the percentage of the islet consisting of beta cells by the pancreas weight.

### Islet isolation and insulin secretion

Intact pancreatic islets were isolated from CTRL (non-Dox-treated) and skmSTX4tg (Dox-treated) mice using collagenase digestion and separation from acinar tissue and debris on Ficoll gradients, followed by hand-picking under a stereomicroscope^55^. Islets were cultured overnight in RPMI 1640 medium (containing 10% FBS, 100 U/ml penicillin, and 100 µg/ml of streptomycin). On the following day, the islets were incubated in 500 μ1 Krebs-Ringer-Bicarbonate-HEPES buffer (KRBH)/Eppendorf tube (25 islets/condition) without glucose for 1 hour, followed by incubation in low (1 mM) and high (20 mM) glucose for 30 min. Insulin assays of the supernatant were performed using a BioTek Synergy (Winooski, VT, USA) HTX Multi-Mode Plate Reader and the Mouse Insulin ELISA kit (Mercodia #10-1247-01; Winston Salem, NC, USA).

### Metabolic caging and body composition analysis

Male mice were metabolically phenotyped during the feeding study, starting at 8 weeks of age (after the chow diet), 18 weeks (after the HFD), or 24 weeks of age (after continuation of the HFD without or with STX4 induction). Mice were weighed, then individually housed in metabolic cages (Phenomaster; TSE Systems, Bad Homburg, Germany) with *ad libitum* access to food and water for the duration of the experiment. The mice were acclimatised to the cages for 1 day and experimental datasets were collected for the next 24-48 hours. The lights were on from 6:00 to 18:00. The following parameters were monitored: food intake, water consumption, oxygen intake, carbon dioxide production, and ambulatory activity. AUC for RER was calculated with 0.7 set to the y-axis, while EE y = 0. AUC was calculated using the area under the curve parameter in Graphpad prism software. Animals were observed daily and body weight was evaluated at the conclusion of the experiment. Whole-body composition (fat and lean tissues) was determined using quantitative magnetic resonance technology (EchoMRI 3-in-1; Echo Medical Systems, Houston, TX).

### Extracellular flux analysis (Seahorse)

Flexor digitorum brevis (FDB) muscle was extracted from both legs of 24-week-old HFD-fed male mice (skmSTX4tg HFD and HFD + STX4) and dissociated in 4 mg/ml collagenase (Sigma, #C-0130) for 2 h. After dissociation, the muscle fibres were cleaned and transferred into immersion media. The myofibers (100 µl/well) were plated in a 24-well Agilent Seahorse eXF24 plate with 10 µl Matrigel in every well. The cells were left to adhere overnight and were analysed in the Agilent extracellular flux analysis machine (XFe24) after treatment with oligomycin (4 μM), FCCP (1 μM), pyruvate (10 μM), and rotenone antimycin A (0.5 μM) at time intervals to assess mitochondrial function. The cells were lysed with 1% NP-40 lysis buffer and the OCR was normalised to protein content. The data were analysed using a wave 2.6.0 software (Agilent, Santa Clara, CA). The results are derived from independent assays of 3 mice and normalised to baseline OCR.

### Citrate synthase activity measurement

TA or whole-hindlimb muscle was homogenised (300 µl TA, 2 ml HL) in ice-cold buffer (50 mM Tris, pH 7.0, 150 mM KCl), and the protein concentration was determined using the BCA Protein Assay (Thermo Scientific). Citrate synthase activity was measured by coupling conversion of oxaloacetate to acetyl-CoA with reduction of 5,5′-dithiosbis (2-nitrobenzoic acid) (DTNB) (Sigma-Aldrich). Protein (20 μg) was added to a 150 μl reaction mix (0.1 mM DTNB, 0.25% Triton X-100, 0.5 mM oxaloacetate and 0.25 mg/ml acetyl-CoA in 0.1 M Tris, 7.4) and product formation was measured at 412 nm over 300 s on a Tecan Infinite M200 PRO plate reader. Results were calculated from a constant rate and an extinction coefficient (εB) 13.6 mM-1•cm-1. Assays were repeated in duplicate, and 2 separate reactions were conducted, at 3 min and 4.5 min^56^.

### Mitochondrial copy number evaluation

DNA was isolated from mouse whole hindlimb, and then analysed by quantitative PCR using SYBR green (Bio-Rad #170-8880) and processed on QuantStudio3 (Applied Biosystems). The abundance of mitochondrial-encoded cytochrome oxidase I subunit (COX1) was compared with the nuclear 18 S rRNA to give the mitochondrial DNA abundance. The mouse COX1 primers were 5′-ACCATCATTTCTCCTTCTCCTA-3′ and 5′-TAGATTTCCGGCTAGAGGTG-3′ and the mouse 18 S primers were 5′-TGGCTCATTAA ATCAGTTATGGT-3′ and 5′-GTCGGCATGTATTAGCTCTAG-3′, as described^57^.

### Transmission electron microscopy

Mouse tissues were dissected and immediately fixed with 2.5% glutaraldehyde, 4% paraformaldehyde in 0.1 M sodium cacodylate buffer (Na(CH_3_)2AsO2 •3H2O), pH 7.2, at 4°C. Standard tissue sample preparation for TEM was followed including post-fixation with osmium tetroxide, serial dehydration with ethanol, and embedment in Eponate^58^. Ultra-thin sections (~70 nm thick) were acquired by ultramicrotomy, post-stained, and examined on an FEI Tecnai 12 transmission electron microscope equipped with a Gatan Ultrascan 2 K CCD camera.

### Immunogold staining

Mouse muscle tissues were dissected and immediately fixed with 0.2% glutaraldehyde, 4% paraformaldehyde in phosphate-buffered saline (PBS), pH 7.4, at 4 °C. The tissues were then sectioned into 150-µm-thick sections using a Leica Vibratome. Pre-embedding immune-labelling was performed on the vibratome sections using rabbit anti-STX4 primary antibody (Millipore Sigma #AB5330) and Nanogold® anti-rabbit IgG (Nanoprobes) diluted 1:100 in PBS for 1.5 h. HQ Silver enhancement kit (Nanoprobes) was used to further develop the Nanogold® staining. The vibratome sections were then processed following standard sample preparation for TEM and examined as described above.

### Mitochondrial area analysis

The mitochondrial area was calculated using the ‘measure’ macro in ImageJ version 1.45 s software. Four to six images per mouse at 6500X magnification were counted, and then converted to mm^2^ by dividing by the number of pixels of the scale bar. Three to six mice were counted per condition.

### Conditioned media assay

L6 GLUT4myc cells and INS-1 832/13 cells were authenticated by the cell morphology using microscopy. Low passages of cells were kept frozen. L6 skeletal muscle myoblasts were transfected with, pCDNA and pCDNA STX4 for 48 h and media was harvested. Myoblast media (250 μl), plus 250 μl low glucose (2.5 mM) and low serum (2.5%) RPMI (INS-1 derived 832/13 cell) media were mixed (equal ratios) and incubated with INS-1 832/13 cells (provided by Dr. Christopher Newgard (Duke University Medical Center, Durham, NC) for 16 hours. The media was washed after 16 hours and incubated in Krebs-Ringer bicarbonate buffer (KRBB) for 1 h. Further, the cells were incubated in 1 mM or 20 mM glucose-containing KRBB for 30 min and the supernatants were collected for GSIS. Insulin released into the media was assessed by rat insulin ELISA kit.

### Muscle mitochondrial fractionation assay

Isolation of mouse skeletal muscle mitochondrial was performed as previously described^59^. Briefly, total hindlimb muscles from C57BL/6 mice were minced on ice in Buffer A (220 mM mannitol, 100 mM sucrose, 100 mM Tris-HCl, 1 mM EGTA, pH 7.4) and transferred to centrifuge tubes using 5 ml HM (250 mM sucrose, 100 mM Tris-HCl, 2 mM EGTA, pH 7.4). Two-stage polytron homogenization and centrifugation were performed as published^60^. The mitochondrial pellet was resuspended in Buffer B (220 mM mannitol, 70 mM sucrose, 20 mM Tris- Cl, 0.02 mM EDTA, 0.2% fatty acid-free BSA, pH 7.4). Protein concentration was determined by Micro BCA Protein Assay (Thermo Fisher Scientific, Rockford, IL, USA).

### Co-immunoprecipitation

Three mg of mouse whole-hindlimb extract was immunoprecipitated by incubating the lysate with 3 µg of STX4 antibody (Millipore Sigma, #AB5330) overnight rotating at 4 °C, followed by incubation with 80 µl of Protein-G Agarose beads for 2 h, rotating at 4 °C. Immunoprecipitated proteins were washed three times with lysis buffer on ice and eluted with SDS sampling buffer containing DTT. Eluted proteins were resolved by 10% SDS-PAGE hand-cast gel overnight at 7 V, and then transferred the next day to PVDF membranes activated with methanol and rapidly rinsed in TBS, pH7.4. The gel was transferred overnight, at which time Ponceau S staining was used to evaluate protein loading, and immunoblotting was conducted according to the previously stated method. Mouse muscle mitochondrial fractions (0.5 mg protein per reaction) was incubated with 0.5 μg of STX4 antibody (Millipore Sigma) overnight at 4 °C. The mixtures of protein and antibody were immunoprecipitated by incubating 80 μl of Protein-G Agarose beads for 2 h at 4 °C. The complex was eluted with SDS sample buffer containing DTT and proteins resolved by 12% SDS-PAGE. The gel was transferred to PVDF followed by immunoblotting.

### Statistics

All data are presented as mean ± SEM. Student’s t-test was used for single comparisons, and ANOVA was used for multiple comparisons. Tukey’s post hoc method was used for individual comparisons after one-way ANOVA. *P* < 0.05 was considered significant using GraphPad Prism software, version 7.02 (unless otherwise specified).

### Reporting summary

Further information on research design is available in the Nature Research Reporting Summary linked to this article.

## Supplementary information

Supplementary Information

Reporting Summary

## Data Availability

All data generated or analysed during this study are included in this published article (and its supplementary information files). Source data are provided with this paper.

## Source data

Source Data
