# Supplementary material for: Enrichment of the exocytosis protein STX4 in skeletal muscle remediates peripheral insulin resistance and alters mitochondrial dynamics via Drp1
Source: Nat Commun. 2022 Jan 20;13:424. doi: 10.1038/s41467-022-28061-w (PMC8776765; doi:10.1038/s41467-022-28061-w)
Supplement: Supplementary file 1 — Supplementary Information [file 41467_2022_28061_MOESM1_ESM.pdf]

Supplementary Information

Enrichment of the exocytosis protein STX4 in skeletal muscle remediates peripheral insulin resistance and alters mitochondrial dynamics via Drp1

Karla E. Merz[1,2*], Jinhee Hwang[1*], Chunxue Zhou[1], Rajakrishnan Veluthakal[1], Erika M. McCown[1], Angelica Hamilton[1], Eunjin Oh[1], Wenting Dai[1], Patrick T. Fueger[1,3], Lei Jiang[1], Janice. M. Huss[1] and Debbie C. Thurmond[1¥].

[1]Department of Molecular & Cellular Endocrinology, Arthur Riggs Diabetes and Metabolism Research Institute, City of Hope, Duarte, CA.

[2]Irell and Manella Graduate School of Biological Sciences, City of Hope, Duarte, CA. Present address, Amgen, Thousand Oaks, CA.

[3]Comprehensive Metabolic Phenotyping Core, Beckman Research Institute of City of Hope, Duarte, CA

[4]Present address: Washington University School of Medicine, St. Louis, MO.

*Authors contributed equally; [¥]Corresponding author (dthurmond@coh.org)

Table of Contents:

Supplementary Methods S3-5
Supplementary Figure 1 ....S6-7
Supplementary Figure 2 ....S8-9
Supplementary Figure 3 ....S10
Supplementary Figure 4 ....S11-12
Supplementary Figure 5 ....S13
Supplementary Figure 6 ....S14-16
Supplementary Figure 7..S17-18

Supplementary Table 1 ………………………………………………………………..S19
Supplementary Table 2 ………………………………………………………………..S20
Supplementary Table 3 ………………………………………………………………..S21
Supplementary Table 4 ………………………………………………………………..S22
References ……………………………………………………………………………...S23

Supplementary Methods

Plasma metabolite analysis. Blood was collected from mice that were fasted for 16 hours. Plasma was separated after centrifugation and used to assess cholesterol (Sigma #MAK043), NEFA (WAKO Diagnostics, NEFA HR2), triglycerides and free glycerol (Sigma #TR0100), adiponectin (Millipore #EZMADP-60K), insulin (Insulin RIA kit, Millipore #SRI-13K), glucagon (Millipore #GL-32K), leptin (Sigma #RAB0334) and adiponectin (Millipore, #EZMADP-60K), according to the manufacturers' instructions.

Extracellular flux analysis (Seahorse) for L6 GLUT4myc cells. L6 GLUT4myc skeletal muscle cells were seeded at 3,000 cells in 24-well Agilent Seahorse eXF24 plates. According to the manufacturer's instruction, cells were transfected with siSTX4 (100 nM) or siCON using RNAiMAX (Invitrogen, Carlsbad, CA, USA) for 24 h, and washed with PBS to remove any free complex. Then, the cells were sequentially transfected with mouse DRP1 or the control plasmid DNA using L6 Cell Avalanche™ Transfection Reagent and then further cultured for 48 h. The cells were starved with XF base medium DMEM, pH 7.4 (Agilent Technology, cat# 103575-100) supplemented with 5.5 mM glucose, 2 mM glutamine, and 1 mM sodium pyruvate, for 1 h in a non-CO2 incubator at 37°C before the assay. OCR was measured by adding oligomycin (1 μM), Carbonyl cyanide-p-trifluoromethoxyphenylhydrazone (FCCP, 2 μM), and Rotenone and antimycin A (RA, 0.5 μM). Following the measurement, OCR was normalized to protein content within the culture wells of the Seahorse plate.

Stable isotope tracing. Stable isotope tracing experiments to determine isotope distributions in soluble metabolites were performed as described[1,2]. Glucose labelled with ^{13}C was purchased from

Cambridge Isotope Laboratories (MA, USA). L6 skeletal muscle myoblasts were grown in 60 mm dishes until 80% confluent, and transfected with rSTX4 and Ctrl plasmid DNA using L6 Cell Avalanche™ Transfection Reagent for 48 h. Then, cells were cultured with ^{13}C-labeled MEMα medium containing 10% FBS for 6 hours. Intracellular metabolites were extracted in a 1:1 mixture of methanol and water, and samples were processed and analysed on an Agilent 7890B gas chromatograph (Agilent, CA, USA).

PKA activity assay. PKA activity was measured using the PKA Colorimetric Activity Kit (Invitrogen, #EIAPKA), following the manufacturer's protocol, using 40 μl of sample. Sample, kit standard and 10 μl of ATP were co-incubated in 96-well plates at 30°C for 90 min while shaking. Wells were washed with kit buffer, then antibodies added for 1 h, then substrate was added to each well for 30 min and reaction stop solution added. Activity was measured at 450 nm using a Synergy HTX multi-mode reader. A standard curve was generated by a four-parameter logistic regression, and the PKA activity was calculated using GraphPad Prism 8.0 software.

2-Deoxyglucose uptake assay in L6 GLUT4myc cells. Differentiated myotubes were serum starved for 50 minutes in FCB buffer (125 mM NaCl, 5 mM KCl, 1.8 mM $CaCl_2$, 2.6 mM $MgSO_4$, 25 mM HEPES, 2 mM pyruvate, 2% BSA) and then stimulated with insulin at 100 nM for 10 min. The cells were then treated with 2-deoxy [1,2,-^{3}H] glucose (Perkin Elmer, Waltham, MA) to initiate glucose uptake and after 5 min, the cells were washed 3 times with ice-cold PBS to terminate the reaction. The cells were lysed by incubation in 250 μl of 1N NaOH for 1 hour, after which 150 μl of sample was transferred into 5 ml scintillation fluid for quantitation of [^{3}H]. The

remaining sample was used for BCA protein analysis. Samples were only read after 24 hours of incubation in the scintillant, and once read; the data was normalised to protein concentration.

Quantitative PCR analysis of mitochondrial dynamics genes. Total RNA from whole hindlimb skeletal muscle was isolated using a Tri Reagent® (Sigma-Aldrich, Cat# 93289) according to the manufacturer's instructions. Fifty nanograms per reaction was used for real time-qPCR using the QuantiTect SYBR Green RT-PCR Kit (Qiagen, cat# 204243) under the following conditions: 40 cycles, at 94˚C for 15 s, 52˚C for 30 s, and 72˚C for 30 s. Relative gene expression was normalized to HPRT by using the ΔΔCT method. Primer sequences used for mitochondrial dynamics genes were listed here: DRP1, for: 5'-CGGTTCCCTAAACTTCACGA-3', rev: 5'-GCACCATTTCATTTGTCACG-3'; MFN1, for: 5'-TTGCCACAAGCTGTGTTCGG-3', rev: 5'-TCTAGGGACCTGAAAGATGGGC-3'; MFN2, for: 5'-GGGGCCTACATCCAAGAGAG-3', rev: 5'-GCAGAACTTTGTCCCAGAGC-3'; Fis1, for: 5'-GCCTGGTTCGAAGCAAATAC-3', rev: 5'-CACGGCCAGGTAGAAGACAT-3'; TFAM, for: 5'-GAAGGGAATGGGAAAGGTAGA-3', rev: 5'-AACAGGACATGGAAAGCAGAT-3'; ATG7, for: 5'-TTTCTGTCACGGTTCGATAATG-3', rev: 5'-TGAATCCTTCTCGCTCGTACT-3'; HPRT: for: 5'-AAGCCTAAGATGAGCGCAAG-3', rev: 5'-TTACTAGGCAGATGGCCACA'3'.

Supplementary Figure 1

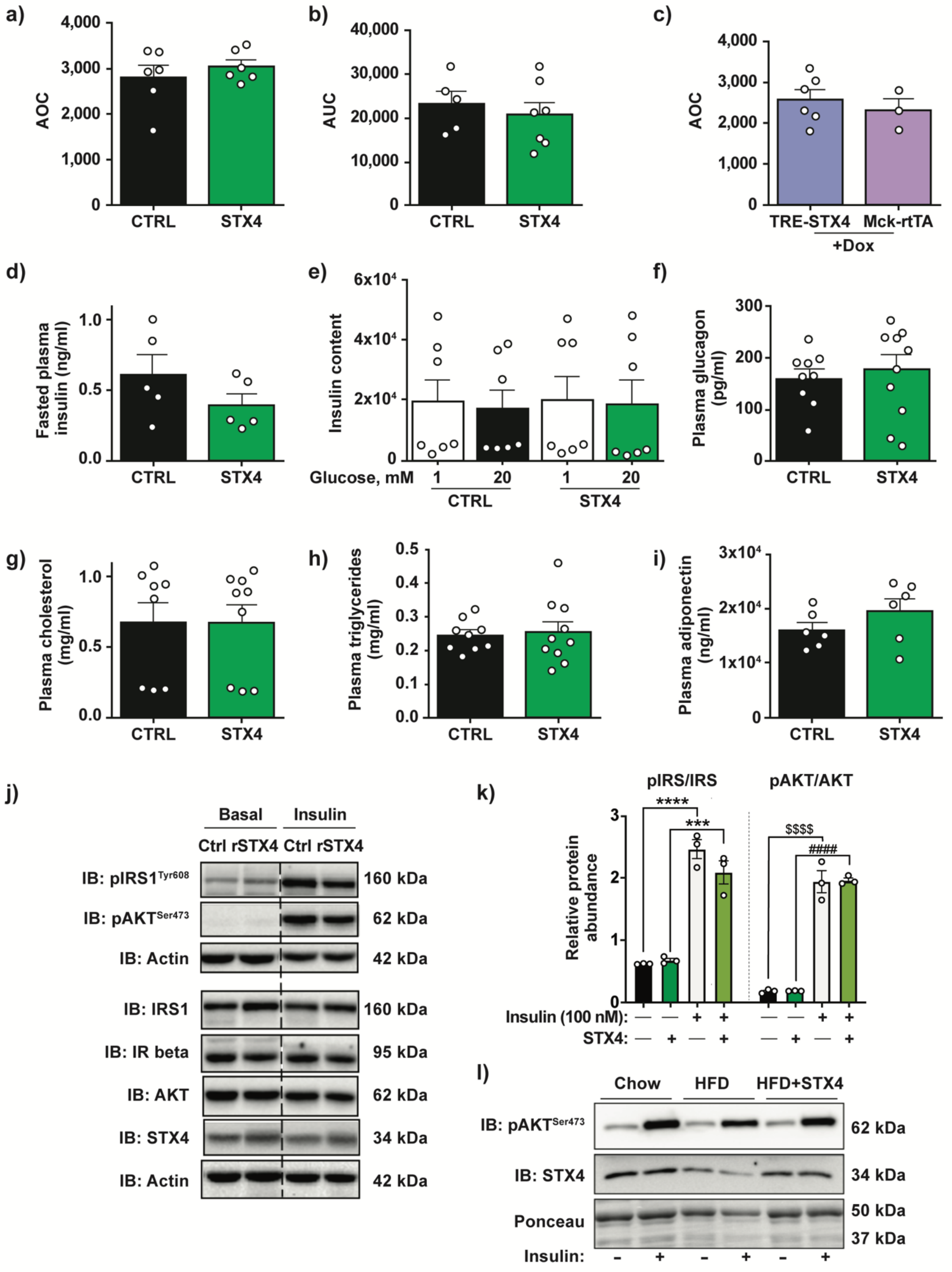

Supplementary Figure 1: Phenotypic data from chow-fed CTRL and skeletal muscle-specific STX4 enriched mice. *a)* Male intraperitoneal insulin tolerance test (IPITT) results, represented as area over the curve (AOC), (CTRL, n=6; STX4, n= 6). *b)* Male intraperitoneal glucose tolerance test (IPGTT) results, represented as area under the curve (AUC), (CTRL, n=5; STX4, n= 7). *c)* IPITT AOC results of single transgenic (STX4-TRE or Mck-rtTA) female mice provided with 2 mg/ml doxycycline (Dox) in water, (STX4-TRE, n=6; Mck-rtTA, n= 3). *d)* Plasma insulin levels in female mice fasted for 16 h, (CTRL, n=5; STX4, n= 5). *e)* Islet insulin content (n=7 mice/group). *f-i)* Plasma metabolites assessed from female mice fasted for 16 h. f, CTRL, n=9, STX4, n= 10; g, CTRL, n=8, STX4, n= 9; h, CTRL, n=9, STX4, n= 10; i, CTRL, n=6, STX4, n= 6. Error bars in (a-i) denote mean±SEM. No significant differences were detected. *j)* Insulin signalling in L6 myoblasts transiently transfected to overexpress STX4 or an empty vector control (CTRL), treated with or without insulin (100 nm, 10 min). Representative of three independent sets of cell passages. The vertical dashed line indicates splicing of lanes from within the same gel exposure. *k)* Bar graph quantitation of $pAKT^{Ser473}$/total AKT and pIRS1/total IRS1, corresponding to *(j)* n=3 biologically independent replicates. Error bars denote mean±SEM. *l)* $pAKT^{Ser473}$ in chow, high-fat diet (HFD) and HFD+STX4 mouse skeletal muscle lysates (n=3 per group). Statistics were calculated using one-way ANOVA with Tukey's post-hoc test. $pIRS^{Tyr608}$/IRS; ****p<0.0001, Ctrl Insulin versus Ctrl basal, ***p=0.0002, rSTX4 Insulin versus rSTX4 basal. $pAKT^{Ser473}$/AKT; $^{\$\$\$\$}$P<0.0001, Ctrl Insulin versus Ctrl basal, $^{\#\#\#\#}$P<0.0001, rSTX4 Insulin versus rSTX4 basal by One-way ANOVA with Tukey's post-hoc test. Source data are provided as a Source Data file.

Supplementary Figure 2

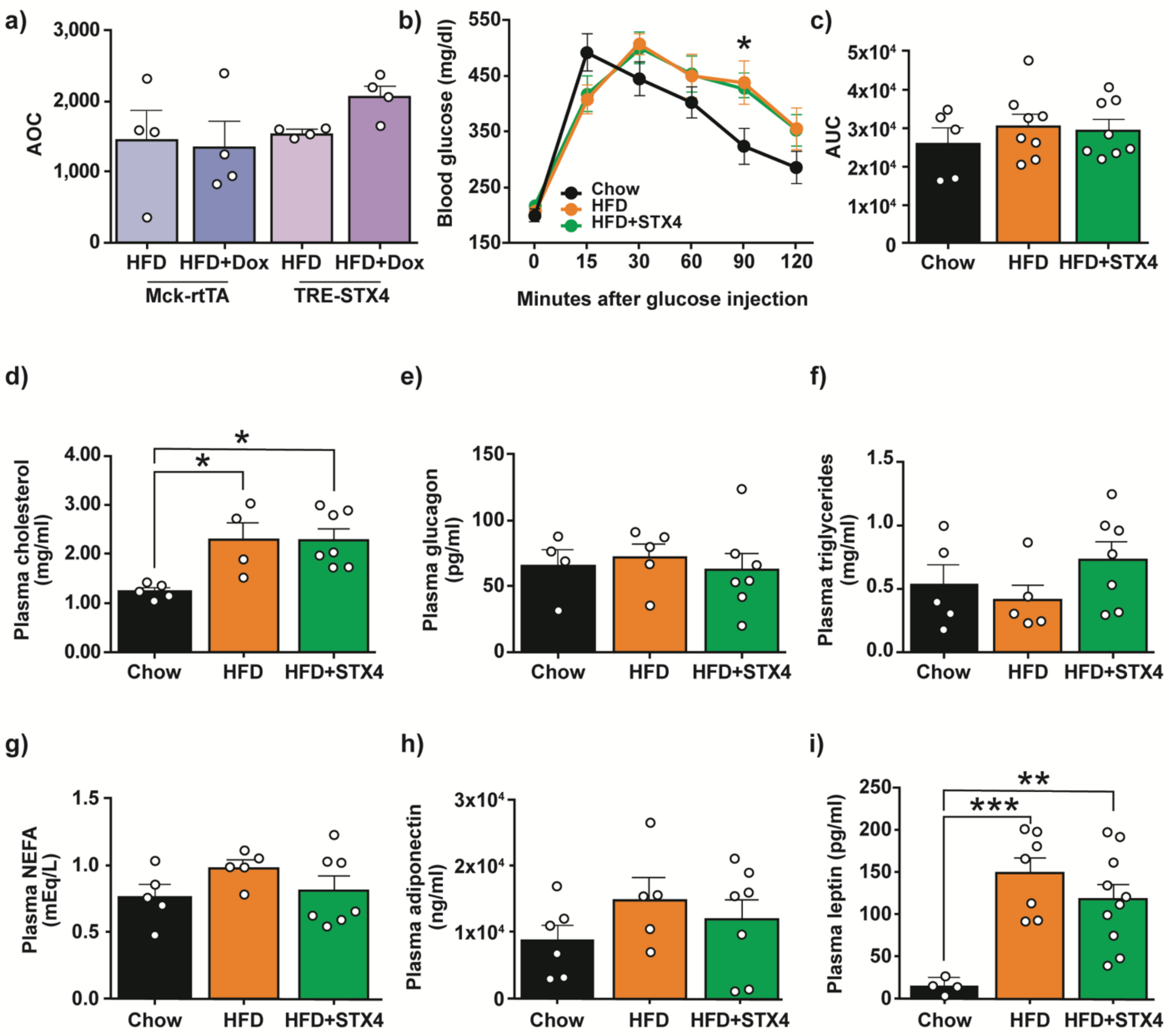

Supplementary Figure 2: HFD IPGTT and plasma metabolites of male mice. *a)* IPITT AOC quantitation for HFD and HFD+Dox single transgenic (TRE-STX4 or Mck-rtTA) male mice, n=4/ group. *b-c)* IPGTT results from 23-week-old male mice fasted for 6 h: chow (black circles, n=5), HFD (orange circles, n=8) or HFD+STX4 mice (green circles, n=8). The bar graph shows data expressed as AUC, no significant differences were observed between HFD and HFD+STX4 mice. *d-i)* Plasma metabolites of male mice fasted for 16 h; d, Chow, n=5; HFD, n= 4; HFD+STX4, n=7; e,

Chow, n=4; HFD, n= 5; HFD+STX4, n=7; f, Chow, n=5; HFD, n= 5; HFD+STX4, n=7; g, Chow, n=5; HFD, n=5; HFD+STX4, n=7; h, Chow, n=6; HFD, n=5; HFD+STX4, n=7; i, Chow, n=4; HFD, n=7; HFD+STX4, n=10. Error bars in (a-i) denote mean±SEM. Statistics were calculated using one-way ANOVA with Tukey's post-hoc test, d, *p=0.0264, HFD versus Chow, *p=0.0107, HFD+STX4 versus Chow; g, ***p=0.009 HFD versus Chow, **p=0.0060 HFD+STX4 versus Chow. Source data are provided as a Source Data file.

Supplementary Figure 3

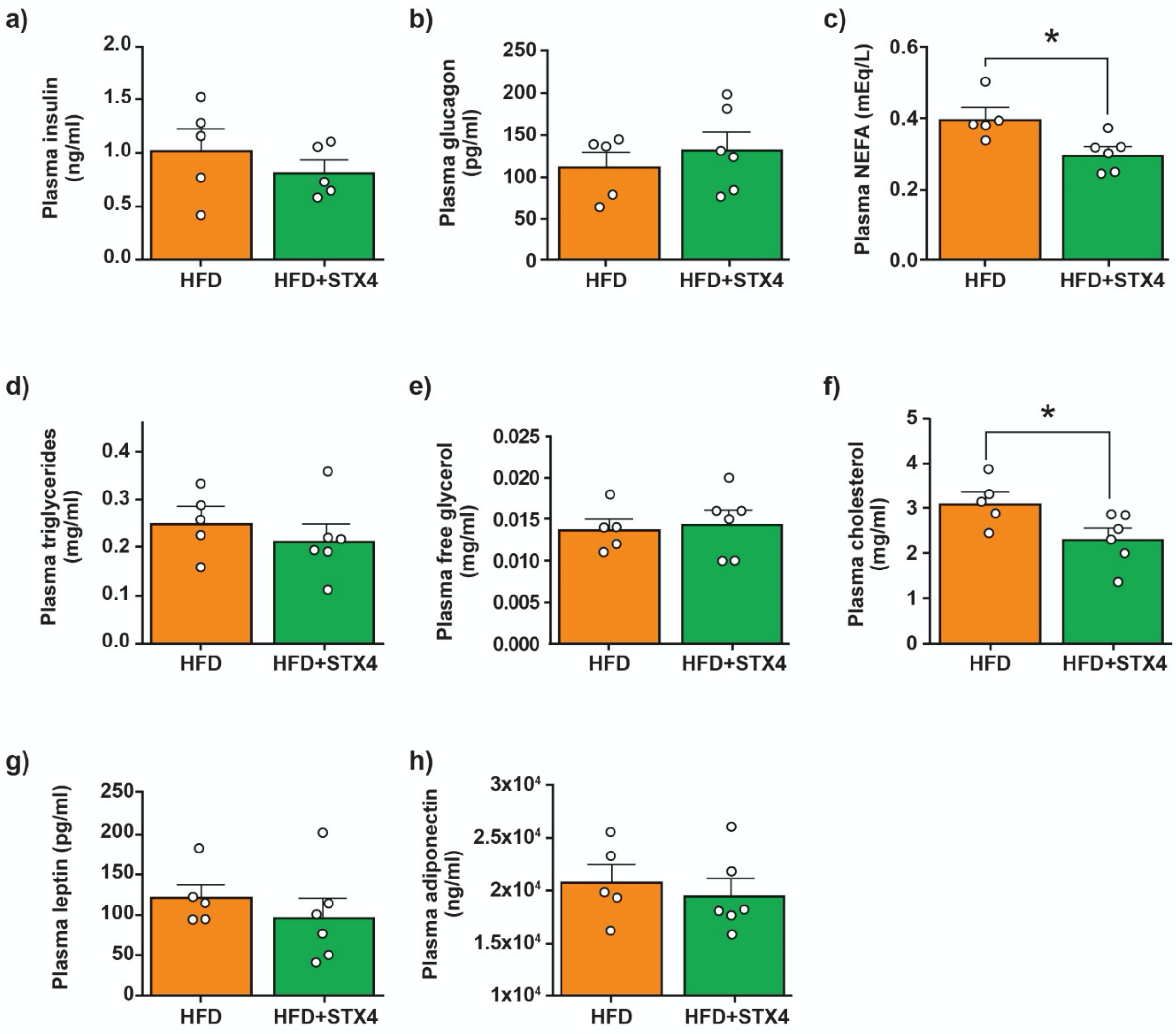

Supplementary Figure 3: Plasma metabolites from male mice fasted for 6 hours. *a-h)* Plasma metabolites from male mice fasted for 6 h (HFD, n=5, HFD+STX4, n=6). Error bars in (a-h) denote mean±SEM. Statistics were calculated using unpaired two-tailed t-test, c, *p=0.0149; f, *=0.0390.

Supplementary Figure 4

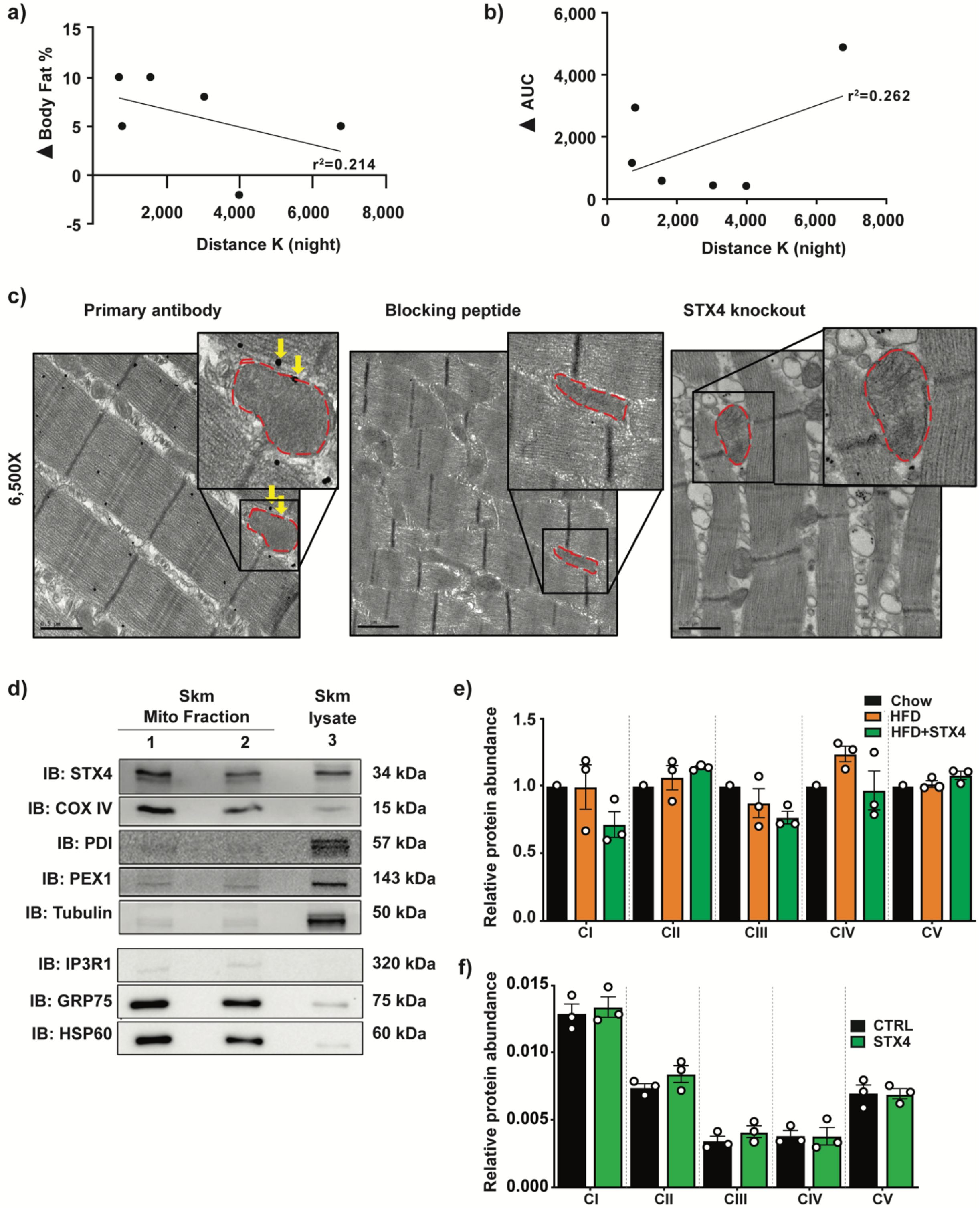

Supplementary Figure 4: Regression analysis of Distance K and validation of immunogold STX4 antibody. *a)* HFD+STX4 night Distance K values were plotted against change in body fat percentage with STX4 induction (p value= 0.36), and *b)* change in insulin sensitivity for each male mouse (AUC, p value = 0.30). No correlation was observed between variables. *c)* Electron microscopy immunogold labelling validation using anti-STX4 blocking peptide. Two corresponding sections from the same tibialis anterior (TA) muscle were sectioned, and primary STX4 antibody was used on one section, whereas primary STX4 antibody mixed at a 1:1 ratio with anti-STX4 blocking peptide was used on the subsequent section. Specificity of the labelling was further tested by comparison with skeletal muscle from skmSTX4-iKO mice. Sections were assessed in a blinded fashion. Bar=0.5 μm; yellow arrows indicate STX4 immunogold labelling, and the red dashed line indicates the perimeter of the mitochondrion (n=3). *d)* STX4 abundance in skeletal muscle mitochondrial fractions vs. whole skeletal muscle lysate, using mitochondria isolated from hindlimb; the whole skeletal muscle (skm) lysate served as a positive control for protein detection by these antibodies (COX IV, mitochondrial marker; PDI, endoplasmic reticulum marker; PEX1, proteasomal marker; Tubulin, cytosolic marker) from three WT mice. Additional markers HSP60, GRP75 and IP3R1 were evaluated to assess the same fractions for a known mitochondrial membrane protein, and mitochondrial associated membrane proteins (MAM), respectively (n=3). *e)* Quantitation of mitochondrial electron transport chain complex I-V (CI-CV) protein abundances in HFD (orange) vs. HFD+STX4 (green) mice (n=3 mice/group; no significant differences detected by one-way ANOVA); *f)* in chow-fed CTRL vs STX4 mice (n=6 mice/ group), no statistical differences were detected by unpaired two-tailed t-test). Error bars in e and f denote mean±SEM. Source data are provided as a Source Data file.

Supplementary Figure 5

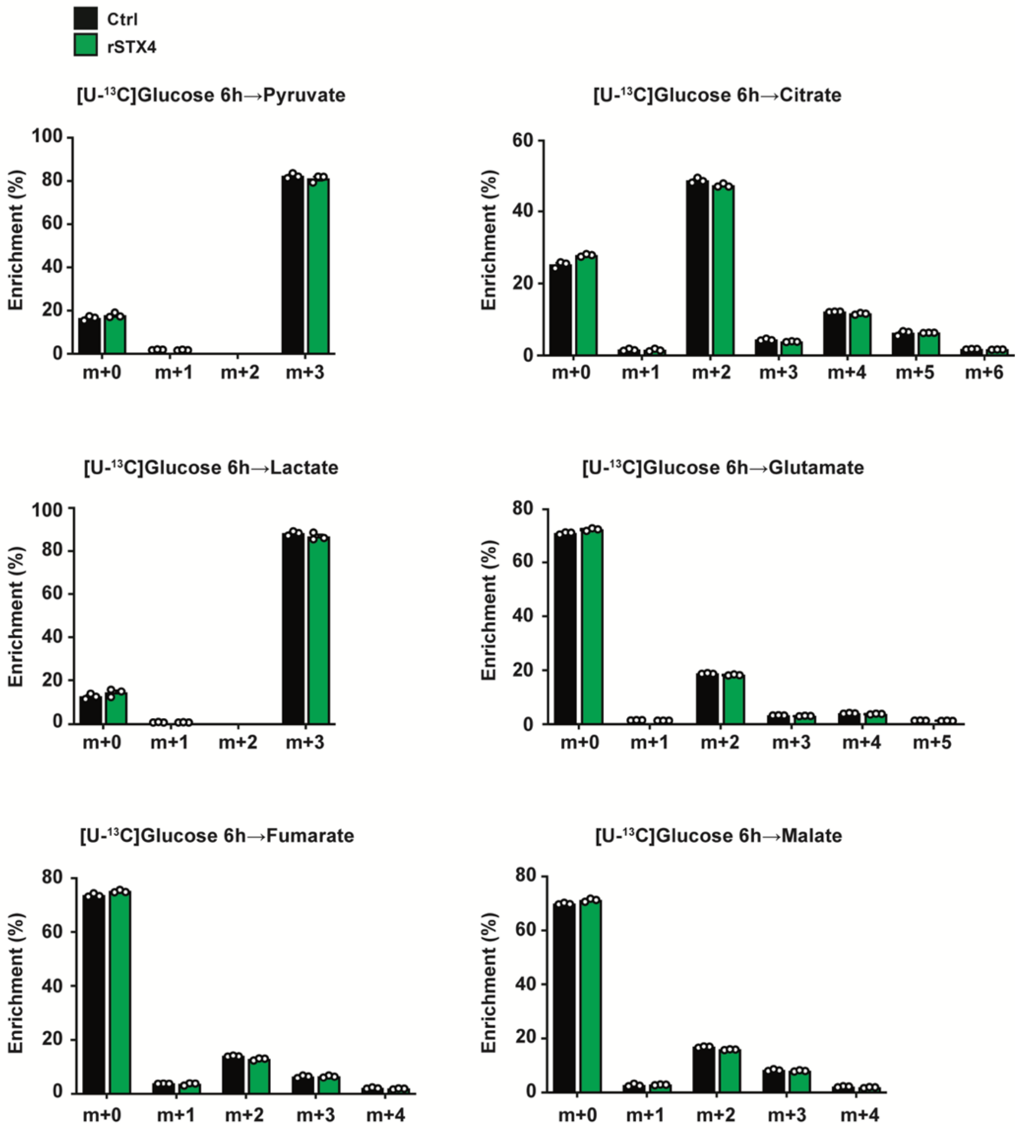

Supplementary Figure 5: STX4 has no effect on tricarboxylic acid (TCA) cycle metabolites

L6 myoblasts were transiently transfected to overexpress rat STX4 (rSTX4) or empty vector (Ctrl). After 48 h, cells were cultured with [U-^{13}C] glucose for 6 h and then evaluated for metabolic enrichment of the following TCA cycle metabolites: pyruvate, citrate, lactate, glutamate, fumarate and malate. Bars represent the mean ± SEM of three independent passages of transfected cell experiments. No significant differences were detected between Ctrl and rSTX4, as determined using unpaired two-tailed t-test. Source data are provided as a Source Data file.

Supplementary Figure 6

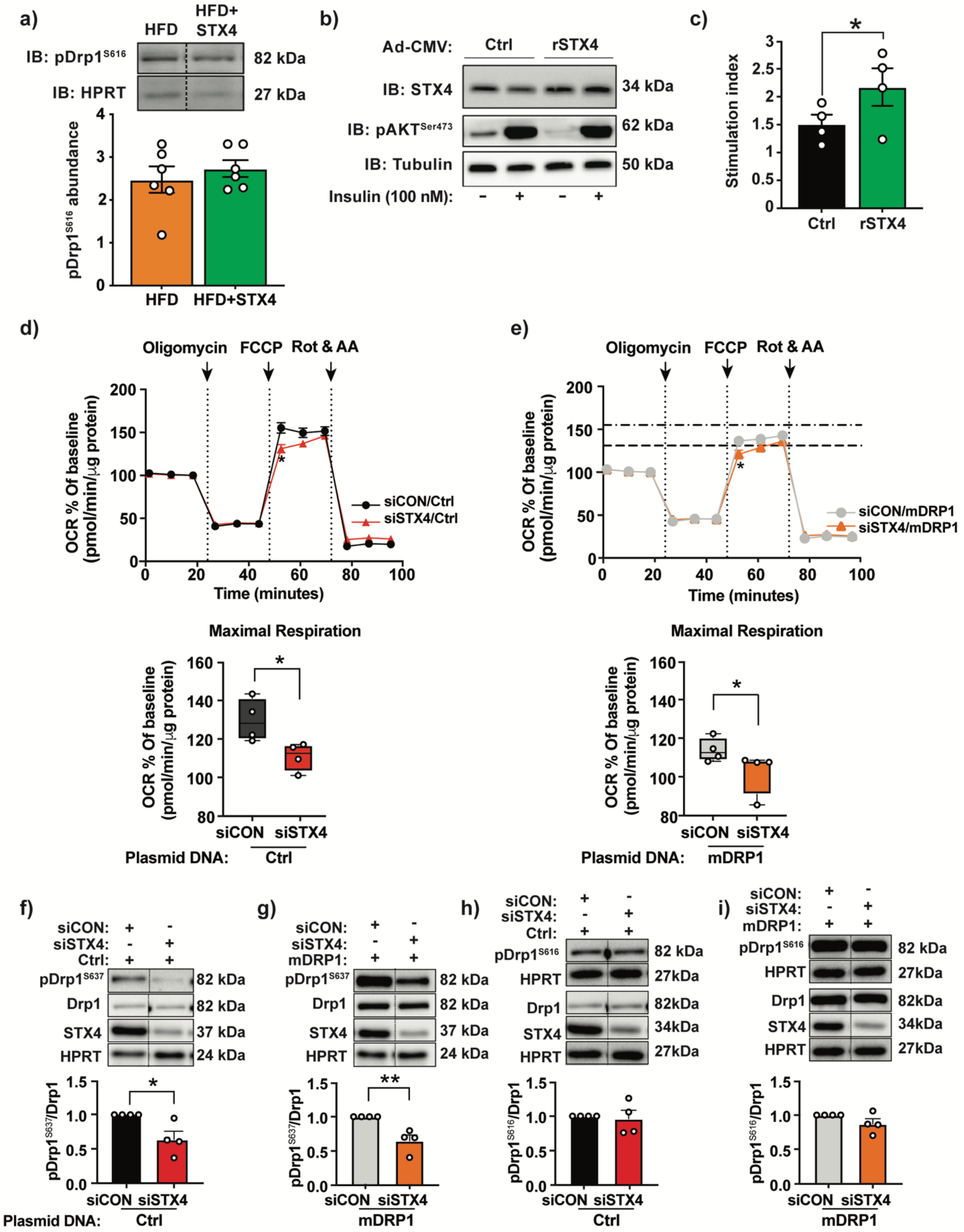

Supplementary Figure 6. Mechanism of STX4 action on mitochondria, metabolism and relationship with Drp1. *a)* pDrp1^{S616} abundance in skeletal muscle of HFD and HFD+STX4 mice (n=6/group). *b)* STX4 enrichment in L6 skeletal muscle myotubes upon transduction with Ad-CMV or Ad-CMV-STX4 (n=4). *c)* Insulin-stimulated 2-deoxyglucose uptake in L6 myotubes with STX4 enrichment, n=4 biologically independent replicates. *p=0.0392 by unpaired two-tailed t-test . *d)* Top panel: Oxygen consumption rate (OCR) in L6 myoblasts sequentially transfected with siSTX4 (siSTX4, red line), compared to control (siCON, black line) for 24 h followed by plasmid empty vector control (Ctrl) for 48 h. *p=0.0205 by unpaired two-tailed t-test. Bottom Panel: Corresponding box and whisker quantification of maximal respiration after treatment with FCCP (2 μM). *p=0.0303 by unpaired two-tailed t-test, n=4 using independent passages of cells. *e)* Top panel: OCR of L6 myoblasts sequentially transfected with siSTX4 for 24 h followed by mouse (m)Drp1 for 48 h (orange line), compared to siCON with Drp1 overexpression, (grey line). *p=0.0395 by unpaired two-tailed t-test. Bottom Panel: Corresponding box and whisker quantification of maximal respiration after treatment with FCCP (2 μM); n=4 using independent passages of cells, *p=0.0286 by unpaired one-tailed Mann-Whitney test. Upper dashed line=OCR after FCCP of siCON/Ctrl in panel d), lower dashed line, OCR after FCCP of siSTX/Ctrl in panel d). *d,e)* Box plots indicate median (middle line), 25th, 75th percentile (box) and minimum and maximum (whiskers) (whiskers). *f-i)* pDrp1^{S637}, total Drp1, STX4 and pDrp1^{S616} abundances in L6 myoblasts transfected with siSTX4 or siCON for 24 h followed by sequential transfection with pCDNA3-mDRP1 or Ctrl (vector) plasmid DNA; n=4 independent sets of transfected cells. siCON in each set of independent cell passages was set=1.0 and siSTX4 ± mDrp1 expression data normalized thereto. Vertical dashed line indicates splicing of a lane from within the same gel exposure. *f)* Ratio of pDrp1^{S637} to Drp1 in siSTX4 transfected cells is reduced, compared to siCON.

g) Drp1 overexpressing cells still show attenuated pDrp1^{S637} /Drp1 in STX4 depleted cells, compared with siCON cells. *h-i)* pDrp1^{S616} to Drp1 ratio is similar in siCON and siSTX4 cells under Ctrl and mDrp1-overexpressing conditions. n=4/group. f; *p=0.0233 by unpaired two-tailed t-test, g; **p=0.0060, unpaired two-tailed t-test. Source data are provided as a Source Data file.

Supplementary Figure 7

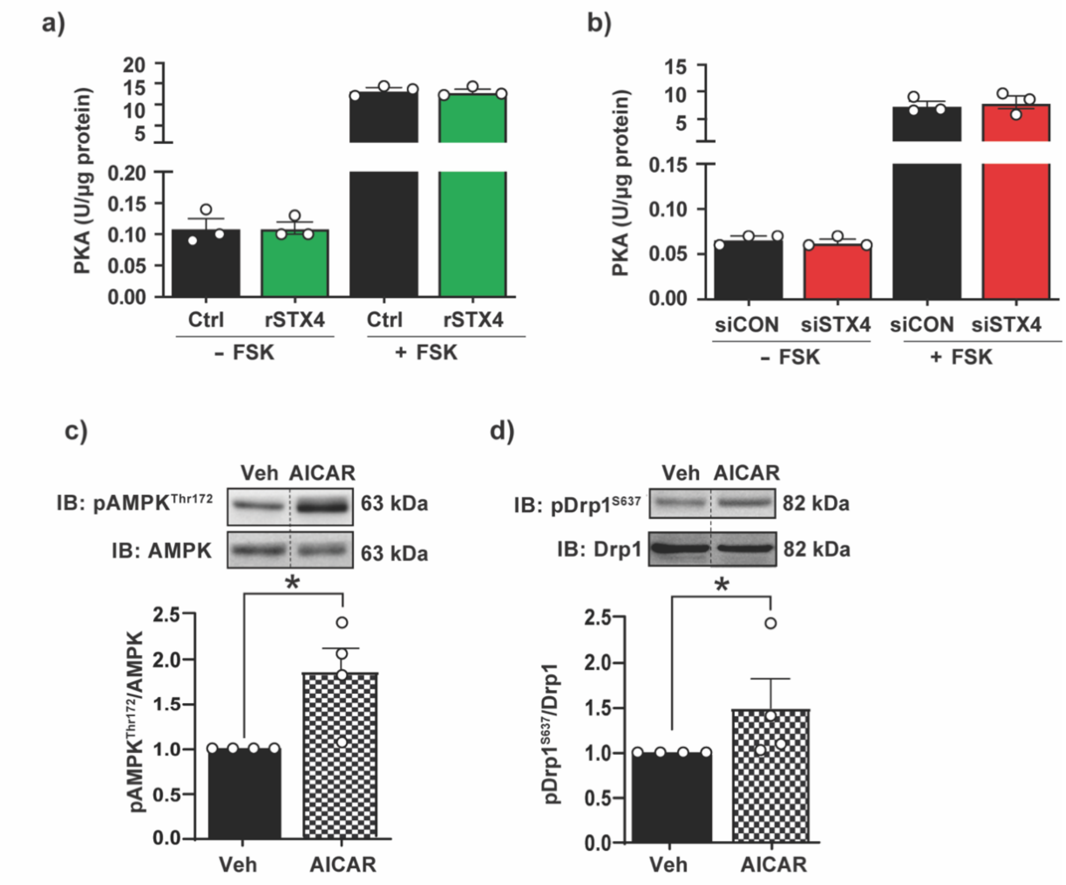

Supplementary Figure 7. STX4 regulates pDrp1S637 in a mechanism involving AMPK but not PKA. *a)* Protein kinase A (PKA) activity in L6 myoblasts overexpressing STX4; n=3 biologically independent replicates. *b)* PKA activity in L6 myoblasts with STX4 knockdown (siSTX4). Forskolin (FSK) was added as a positive control to activate PKA; n=3 biologically independent replicates. *c)* Validation of AMPK activation (pAMPK) by AICAR treatment in L6 myoblasts (*p=0.0264 by unpaired two-tailed t-test) normalised to total AMPK and then to Veh (vehicle) set equal to 1 in each set of independent experiments to determine fold change; n=4 biologically independent replicates. *d)* Validation of AICAR-stimulated phosphorylation of Drp1 at S637 in L6 myoblasts (*p=0.0286 by unpaired two-tailed Mann-Whitney test) normalised to total Drp1 and then to Veh (vehicle) se equal to 1 in each set of independent experiments to

determine fold change; n=4 biologically independent replicates. The vertical dashed line indicates splicing of lanes from within the same gel exposure. Error bars in (a-d) represent mean±SEM. Source data are provided as a Source Data file.

Supplementary Table 1. Tissue and body weights of chow-fed CTRL and STX4 (Dox-induced) skmSTX4Tg female mice.

	CTRL (n=6)	STX4 (n=7)
Body weight (g)	22.8 ± 0.9	22.4 ± 0.9
	Tissue (% body weight)	
Kidney	1.34 ± 0.03	1.37 ± 0.10
Heart	0.59 ± 0.03	0.59 ± 0.08
Spleen	0.45 ± 0.06	0.31 ± 0.06
Pancreas	1.20 ± 0.12	0.98 ± 0.09
Fat	1.22 ± 0.12	0.83 ± 0.17
Muscle	3.04 ± 0.13	3.11 ± 0.11
Liver	4.14 ± 0.12	3.96 ± 0.18
Lungs	0.61 ± 0.04	0.70 ± 0.06
Brain	1.84 ± 0.05	2.05 ± 0.07

Data represent the average ± SEM of 4-6-month-old female mice. No significant differences were detected by unpaired two-tailed t-test. Source data are provided as a Source Data file.

Supplementary Table 2. Tissue and body weights of chow, HFD, and HFD + STX4 male mice.

	Chow (n=4)	HFD (n=6)	HFD+ STX4 (n=7)
Body weight (g)	31.0 ± 0.4	37.7 ± 1.7*	44.1 ± 1.2****
		Tissue (% body weight)	
Kidney	1.36 ± 0.01	1.04 ± 0.04**	1.01 ± 0.06***
Heart	0.61 ± 0.08	0.54 ± 0.05	0.51 ± 0.04
Spleen	0.23 ± 0.03	0.27 ± 0.03	0.26 ± 0.02
Pancreas	0.74 ± 0.07	0.55 ± 0.07	0.68 ± 0.03
Fat	1.90 ± 0.24	5.28 ± 0.46****	6.11 ± 0.29****
Muscle	2.58 ± 0.26	1.70 ± 0.40	1.30 ± 0.25
Liver	4.70 ± 0.24	3.93 ± 0.16*	4.20 ± 0.15
Lungs	0.57 ± 0.05	0.58 ± 0.07	0.57 ± 0.03
Brain	1.22 ± 0.02	1.17 ± 0.15	1.08 ± 0.03

Data represent the average ± SEM of 4-6-month-old male mice. Statistics calculated using one-way ANOVA with Tukey's multiple comparison test; Body weight, *p=0.0189, HFD versus Chow, ****p<0.0001, HFD+STX4 versus Chow; Kidney **p=0.0028, HFD versus Chow, ***p=0.0009, HFD+STX4 versus Chow; Fat, ****p<0.0001, HFD versus Chow, ****p<0.0001, HFD+STX4 versus Chow; Liver, *p=0.0311, HFD versus Chow. Source data are provided as a Source Data file.

Supplementary Table 3. Quantitative PCR analysis of mitochondrial dynamics genes.

Gene	Fold change
Drp1	1.00
MFN1	0.99
MFN2	0.97
Fis1	1.03
TFAM	0.90
ATG7	1.06

Data represent the average ± SEM of whole hindlimb skeletal muscle from six sets of 4-6 month old female skmSTX4tg and littermate control (CTRL) mice normalized to HPRT. No significant differences were detected by unpaired two-tailed t-test. Source data are provided as a Source Data file.

Supplementary Table 4. Antibodies used for immunohistochemistry and co-immunoprecipitation

Antibody	Vendor	Cat#	Lot#	Size	Source	Dilution	IP or WB
**IRS1**	Cell signaling	2390s	7	160	Rabbit	1:1,000	WB
**IR beta**	Abcam	ab137747	GR3277870-05	95	Rabbit	1:5,000	WB
**AKT**	Cell signaling	9272s	28	60	Rabbit	1:1,000	WB
**STX4**	Home-made			34	Rabbit	1:2,000 or 1:5,000	WB
**STX4**	Millipore Sigma	AB5330-200UL		34	Rabbit	1 μg/mg protein	IP
**HPRT**	Abcam	ab10479	GR3227070-1	24	Rabbit	1:1,000 or 1:5,000	WB
**p-IRS1^{Tyr608}**	Millipore	09-432	3260921	160	Rabbit	1: 1,000	WB
**p-IR^{Tyr1362}**	Abcam	ab60946	GR3324684-1	95	Rabbit	1:500	WB
**p-AKT^{Ser473}**	Cell signaling	4060s	25	60	Rabbit	1:1,000	WB
**Actin**	Abcam	ab8227		42	Rabbit	1:10,000	WB
**AMPKα**	Cell signaling	2793		62	Mouse	1:1,000	WB
**p-AMPK^{Thr172}**	Cell signaling	2535			Rabbit	1:1,000	WB
**Drp1**	Abcam	ab56788		82	Mouse	1:1,000	
**p-Drp1^{Ser616}**	Cell signaling	3455S	6	82	Rabbit	1:1,000	WB
**p-Drp1^{Ser637}**	Cell signaling	4867S	4	82	Rabbit	1:1,000	WB
**α-tubulin**	Sigma	T5168		50	Mouse	1:10,000	WB
**COX IV**	Cell signaling	4850	7	17	Rabbit	1:1,000	WB
**PDI**	Cell signaling	2446S		57	Rabbit	1:1,000	WB
**PEX1**	Proteintech	13669-1-AP		143	Rabbit	1:1,000	WB
**IP3R1**	Santa Cruz	sc-271197	D2021	320	Mouse	1:500	WB
**GRP75**	Santa Cruz	sc-133137	B0421	75	Mouse	1:1,000	WB
**HSP60**	Cell signaling	12165	4	60	Mouse	1:1,000	WB
**VDAC1**	Santa Cruz	sc-8829	1070	31	Goat	1:200	WB

WB, western blot. IP, immunoprecipitation.

References:

1. Jiang, L. *et al*. Quantitative metabolic flux analysis reveals an unconventional pathway of fatty acid synthesis in cancer cells deficient for the mitochondrial citrate transport protein. *Metab Eng* **43**, 198-207, doi:10.1016/j.ymben.2016.11.004 (2017).
2. Dai, W. *et al*. Mitochondrial division inhibitor (mdivi-1) decreases oxidative metabolism in cancer. *British Journal of Cancer* **122**, 1288-1297, doi:10.1038/s41416-020-0778-x (2020).
