# Supplementary material for: Enrichment of the exocytosis protein STX4 in skeletal muscle remediates peripheral insulin resistance and alters mitochondrial dynamics via Drp1
Source: Nat Commun. 2022 Jan 20;13:424. doi: 10.1038/s41467-022-28061-w (PMC8776765; doi:10.1038/s41467-022-28061-w)
Supplement: Supplementary file 2 — Reporting Summary [file 41467_2022_28061_MOESM2_ESM.pdf]

nature research

Corresponding author(s): Debbie Thurmond
Last updated by author(s): Dec 1, 2021

Reporting Summary

Nature Research wishes to improve the reproducibility of the work that we publish. This form provides structure for consistency and transparency in reporting. For further information on Nature Research policies, see our Editorial Policies and the Editorial Policy Checklist.

Statistics

For all statistical analyses, confirm that the following items are present in the figure legend, table legend, main text, or Methods section.

n/a	Confirmed	
☐	☒	The exact sample size (n) for each experimental group/condition, given as a discrete number and unit of measurement
☐	☒	A statement on whether measurements were taken from distinct samples or whether the same sample was measured repeatedly
☐	☒	The statistical test(s) used AND whether they are one- or two-sided<br>*Only common tests should be described solely by name; describe more complex techniques in the Methods section.*
☒	☐	A description of all covariates tested
☒	☐	A description of any assumptions or corrections, such as tests of normality and adjustment for multiple comparisons
☐	☒	A full description of the statistical parameters including central tendency (e.g. means) or other basic estimates (e.g. regression coefficient) AND variation (e.g. standard deviation) or associated estimates of uncertainty (e.g. confidence intervals)
☐	☒	For null hypothesis testing, the test statistic (e.g. F, t, r) with confidence intervals, effect sizes, degrees of freedom and P value noted<br>*Give P values as exact values whenever suitable.*
☒	☐	For Bayesian analysis, information on the choice of priors and Markov chain Monte Carlo settings
☒	☐	For hierarchical and complex designs, identification of the appropriate level for tests and full reporting of outcomes
☒	☐	Estimates of effect sizes (e.g. Cohen's d, Pearson's r), indicating how they were calculated

Our web collection on statistics for biologists contains articles on many of the points above.

Software and code

Policy information about availability of computer code

Data collection	Data were collected using the following instruments as indicated in the Methods section:<br>ELISA data collection: BioTek Synergy HTX Multi-Mode Plate Reader (Winooski, VT, USA)<br>Metabolic caging data collection: Phenomaster; TSE Systems, (Bad Homburg, Germany)<br>Whole-body composition data collection: EchoMRI 3-in-1; Echo Medical Systems,(Houston, TX)<br>Extracellular flux analysis data collection:Agilent extracellular flux analysis machine (Santa Clara, CA)<br>Quantitative PCR data collection: QuantStudio3 (Applied Biosystems)<br>Stable isotope tracing data collection: Agilent 7890B gas chromatograph (Santa Clara, CA)<br>Immunoblotting data collection: Bio-Rad ChemiDoc Touch (Hercules, CA)<br>Transmission electron microscopy data collection: FEI Tecnai 12 transmission electron microscope equipped with a Gatan Ultrascan 2K CCD camera
Data analysis	Data analyses were performed using the following software as indicated in the Methods section:<br>Beta cell area data analysis: Keyence BZX-Analyzer software version 1.3.1.1 (Itasca, IL)<br>Mitochondrial area analysis: open-source software Image J version 1.45s (NIH, Bethesda, MD)<br>Extracellular flux analysis data analysis: Wave 2.6.0 (Santa Clara, CA)<br>Statistics: GraphPad Prism version 7.02 (San Diego, CA)

For manuscripts utilizing custom algorithms or software that are central to the research but not yet described in published literature, software must be made available to editors and reviewers. We strongly encourage code deposition in a community repository (e.g. GitHub). See the Nature Research guidelines for submitting code & software for further information.

Data

Policy information about availability of data

All manuscripts must include a data availability statement. This statement should provide the following information, where applicable:

- Accession codes, unique identifiers, or web links for publicly available datasets
- A list of figures that have associated raw data
- A description of any restrictions on data availability

All data generated or analysed during this study are included in this published article (and its supplementary information files). Source data are provided with this paper.

Field-specific reporting

Please select the one below that is the best fit for your research. If you are not sure, read the appropriate sections before making your selection.

[x] Life sciences [ ] Behavioural & social sciences [ ] Ecological, evolutionary & environmental sciences

For a reference copy of the document with all sections, see nature.com/documents/nr-reporting-summary-flat.pdf

Life sciences study design

All studies must disclose on these points even when the disclosure is negative.

Sample size	All sample sizes were included in the figure legends. No statistical methods were applied to predetermine sample size. At least three animals per treatment group or at least three independent replicates for in vitro experiments represents the minimum number animals and attempts for cell culture experiments needed to reach statistical significance ($p < 0.05$) between experimental groups. In mouse studies, a minimum of 3 independent cohorts of 14 or fewer mice per cohort were used.
Data exclusions	Animals were excluded from final tabulation if they showed malocclusion and were culled from the study (Suppl. Table 2). Cell line control samples are included in each passage, and those failing to pass the test for capability to appropriately respond to physiologic stimuli (glucose for beta cells, insulin for muscle cells) are eliminated from the final tabulation. In the current studies, all cell lines passed this basic test and all data were included.
Replication	Experiments were repeated by multiple times across different cohorts of mice and different cell passages (at least three times) and were reproducible. Detailed information on replicates were described in the figure legends. All attempts performed independently were successful.
Randomization	Male HFD mice were intentionally not randomized into treatment groups. The most diseased animals were used with the treatment. This was designed to stringently test the treatment under the most challenging circumstances. To control for co-variates, biological replicates of HFD fed single transgenic mice treated with or without doxycycline were performed. Randomization is not applicable to cell culture experiments.
Blinding	Microscopists were blinded to the identity of the sample type for unbiased imaging and quantitation. For experiments other than the ones mentioned here, no blinding methods were used.

Reporting for specific materials, systems and methods

We require information from authors about some types of materials, experimental systems and methods used in many studies. Here, indicate whether each material, system or method listed is relevant to your study. If you are not sure if a list item applies to your research, read the appropriate section before selecting a response.

Materials & experimental systems

n/a	Involved in the study	
[ ]	[x]	Antibodies
[ ]	[x]	Eukaryotic cell lines
[x]	[ ]	Palaeontology and archaeology
[ ]	[x]	Animals and other organisms
[x]	[ ]	Human research participants
[x]	[ ]	Clinical data
[x]	[ ]	Dual use research of concern

Methods

n/a	Involved in the study	
[x]	[ ]	ChIP-seq
[x]	[ ]	Flow cytometry
[x]	[ ]	MRI-based neuroimaging

Antibodies

Antibodies used	Anti-IRS1: Rabbit monoclonal, Cell signaling, Cat#2390s, Lot#7, 1:1,000<br>Anti-IR beta: Rabbit polyclonal, Abcam, Cat#ab137747, Lot#GR3277870-05, 1:5,000<br>Anti-AKT: Rabbit polyclonal,Cell signaling, Cat#9272s, Lot#28, 1:1,000

Anti-STX4: Rabbit polyclonal, Millipore Sigma, Cat#AB5330-200UL, 1 µg/mg protein
Anti-HPRT: Rabbit polyclonal, Abcam, Cat#ab10479, Lot#GR3227070-1, 1:1,000 or 1:5,000
Anti-p-IRS1Tyr608: Rabbit polyclonal, Millipore Sigma, Cat#09-432, Lot#3260921, 1: 1,000
Anti-p-IRTyr1362: Rabbit polyclonal, Abcam, Cat#ab60946, Lot#GR3324684-1, 1:500
Anti-p-AKTSer473: Rabbit monoclonal, Cell signaling, Cat#4060s, Lot#25, 1:1,000
Anti-Actin: Rabbit polyclonal, Abcam, Cat#ab8227, 1:10,000
Anti-AMPKa: Mouse monoclonal, Cell signaling, Cat#2793, 1:1,000
Anti-p-AMPKThr172: Rabbit monoclonal, Cell signaling, Cat#2535, 1:1,000
Anti-Drp1: Mouse monoclonal, Abcam, Cat#ab56788, 1:1,000
Anti-p-Drp1Ser616: Rabbit polyclonal, Cell signaling, Cat#3455S, Lot#6, 1:1,000
Anti-p-Drp1Ser637: Rabbit polyclonal, Cell signaling, Cat#4867S, Lot#4, 1:1,000
Anti-a-tubulin: Mouse monoclonal, Sigma, Cat#T5168, Lot#4, 1:10,000
Anti-COX IV: Rabbit monoclonal, Cell signaling, Cat#4850, Lot#7, :1,000
Anti-PDI: Rabbit polyclonal, Cell signaling, Cat#2446S, 1 :1,000
Anti-PEX1: Rabbit polyclonal, Proteintech, Cat#13669-1-AP, 1 :1,000
Anti-IP3R1: Mouse monoclonal, Santa Cruz, Cat#sc-271197, Lot#D2021, 1:500
Anti-GRP75: Mouse monoclonal, Santa Cruz, Cat#sc-133137, Lot#B0421, 1:1,000
Anti-HSP60: Rabbit monoclonal, Cell signaling, Cat#12165, Lot#4, :1,000
Anti-VDAC1: Goat polyclonal, Santa Cruz, Cat#sc-8829, Lot#1070, 1:200
Anti-STX4 (Custom-made): Rabbit polyclonal, produced using the mouse 2–23 residue antigenic peptide (Wiseman et al., 2011), 1:2,000 or 1:5,000
Reference for Anti-STX4 (Custom-made)
Wiseman, D. A., Kalwat, M. A., and Thurmond, D. C. (2011) Stimulus-induced S-nitrosylation of syntaxin 4 impacts insulin granule exocytosis. The Journal of biological chemistry 286, 16344-16354

Validation

All antibodies except for Anti-STX4 (Custom-made) used in this study have been extensively used and validated by the manufacturer. All information can be found at the following links:
Anti-IRS1 (Cell signaling, Cat#2390s): https://www.cellsignal.com/products/primary-antibodies/irs-1-59g8-rabbit-mab/2390?site-search-type=Products&N=4294956287&Ntt=2390s&fromPage=plp&_requestid=395842
Anti-IR beta (Abcam, Cat#ab137747): https://www.abcam.com/insulin-receptor-antibody-ab137747.html
Anti-AKT (Cell signaling, Cat#9272s): https://www.cellsignal.com/products/primary-antibodies/akt-antibody/9272?site-search-type=Products&N=4294956287&Ntt=9272s&fromPage=plp&_requestid=396431
Anti-STX4 (Millipore Sigma, Cat#AB5330-200UL):https://www.sigmaaldrich.com/US/en/product/mm/ab5330?context=product
Anti-HPRT (Abcam, Cat#ab10479): https://www.abcam.com/hprt-antibody-ab10479.html
Anti-p-IRS1Tyr608 (Millipore Sigma, Cat#09-432): https://www.sigmaaldrich.com/US/en/product/mm/09432?context=product
Anti-p-IRTyr1362 (Abcam, Cat#ab60946): https://www.abcam.com/insulin-receptor-phospho-y1361-antibody-ab60946.html
Anti-p-AKTSer473(Cell signaling, Cat#4060s): https://www.cellsignal.com/products/primary-antibodies/phospho-akt-ser473-d9e-xp-rabbit-mab/4060?site-search-type=Products&N=4294956287&Ntt=4060s&fromPage=plp&_requestid=396732
Anti-Actin (Abcam, Cat#ab8227): https://www.abcam.com/beta-actin-antibody-ab8227.html
Anti-AMPKa (Cell signaling, Cat#2793): https://www.cellsignal.com/products/primary-antibodies/ampka-f6-mouse-mab/2793
Anti-p-AMPKThr172 (Cell signaling, Cat#2535): https://www.cellsignal.com/products/primary-antibodies/phospho-ampka-thr172-40h9-rabbit-mab/2535
Anti-Drp1 (Abcam, Cat#ab56788): https://www.abcam.com/drp1-antibody-3b5-ab56788.html
Anti-p-Drp1Ser616 (Cell signaling, Cat#3455S): https://www.cellsignal.com/products/primary-antibodies/phospho-drp1-ser616-antibody/3455?site-search-type=Products&N=4294956287&Ntt=3455s&fromPage=plp&_requestid=396930
Anti-p-Drp1Ser637 (Cell signaling, Cat#4867S): https://www.cellsignal.com/products/primary-antibodies/phospho-drp1-ser637-antibody/4867?site-search-type=Products&N=4294956287&Ntt=4867s&fromPage=plp&_requestid=396973
Anti-a-tubulin (Sigma, Cat#T5168): https://www.sigmaaldrich.com/US/en/product/sigma/t5168?context=product
Anti-COX IV (Cell signaling, Cat#4850): https://www.cellsignal.com/products/primary-antibodies/cox-iv-3e11-rabbit-mab/4850
Anti-PDI (Cell signaling, Cat#2446S): https://www.cellsignal.com/products/primary-antibodies/pdi-antibody/2446?site-search-type=Products&N=4294956287&Ntt=2446s&fromPage=plp&_requestid=397030
Anti-PEX1 (Proteintech, Cat#13669-1-AP): https://www.ptglab.com/products/PEX1-Antibody-13669-1-AP.htm
Anti-IP3R1 (Santa Cruz, Cat#sc-271197): https://www.scbt.com/p/ip3r-i-antibody-e-8?requestFrom=search
Anti-GRP75 (Santa Cruz, Cat#sc-133137): https://www.scbt.com/p/grp-75-antibody-d-9?requestFrom=search
Anti-HSP60 (Cell signaling, Cat#12165): https://www.cellsignal.com/products/primary-antibodies/hsp60-d6f1-xp-rabbit-mab/12165
Anti-VDAC1 (Santa Cruz, Cat#sc-8829): https://www.scbt.com/p/vdac1-antibody-c-20?requestFrom=search

STX4 custom antibody not commercially available were validated by Wiseman et al., 2011 and also tested using blocking peptides as well as tissues from knockout mice to show specificity in this study.
Reference: Wiseman, D. A., Kalwat, M. A., and Thurmond, D. C. (2011) Stimulus-induced S-nitrosylation of syntaxin 4 impacts insulin granule exocytosis. The Journal of biological chemistry 286, 16344-16354

Eukaryotic cell lines

Policy information about cell lines

Cell line source(s)

L6GLUT4myc cells were purchased from Kerafast (Cat# ESK202-FP, https://www.kerafast.com/item/840/l6-glut4myc-rat-myoblast-cell-line).

	INS-1 832/13 cells were provided by Dr. Christopher Newgard (Duke University Medical Center, Durham, NC).
Authentication	L6 GLUT4myc cell l and INS-1 832/13 cells were authenticated by the cell morphology using microscopy. Low passages of cells were kept frozen.
Mycoplasma contamination	We routinely test for mycoplasma; all tests are negative, most recent test was in March 2020
Commonly misidentified lines (See ICLAC register)	NO commonly misidentified cell lines were used in the study

Animals and other organisms

Policy information about studies involving animals; ARRIVE guidelines recommended for reporting animal research

Laboratory animals	All animal experiments were conducted in accordance with the NIH Guide for the Care and Use of Laboratory Animals (National Institutes of Health Publication no. 85-23, revised 1996) and approved by the Institutional Animal Care and Use Committees of City of Hope National Medical Center (Duarte, CA, USA; approval #15023). Single transgenic TRE-STX4 mice were generated on the C57BL6J background. Mck-rtTA mice were purchased from the Jackson Laboratory (Bar Harbor, ME, USA) and maintained on the same background. Double transgenic mice were heterozygous for each transgene, obtained from heterozygous matings. Chow-fed mice were housed on Sani-Chip bedding in groups of 3-5 and fed standard maintenance chow (13% of kcal from fat; PicoLab #5053) starting at 4 weeks of age. In the HFD-feeding paradigm, 8-week-old skmSTX4tg mice were fed a custom diet (45% of kcal from fat, principally palmitate based; Research Diets #D01030108) ad libitum for 10 weeks. The most insulin-resistant mice were placed on HFD supplemented with 625 mg Dox (45% of kcal from fat; Research Diets #D17100202) for 4 weeks, whereas the remaining mice fed HFD were maintained on the HFD without Dox. All HFD-fed mice were housed individually on Sani-Chip bedding; 12 h light, 12h dark cycle. STX4 floxed (fl/fl) mice were obtained from Dr. Sidney Whiteheart (University of Kentucky) and crossed with human skeletal actin (HSA)-rtTA/TRE-Cre recombinase positive mice (JAX # 012433) to produce doxycycline-inducible STX4 fl/fl:Cre+ mice (KO, heterozygous for Cre). Mice were treated with 2 mg/kg doxycycline in water to induce STX4 knockout.
Wild animals	No wild animals were used.
Field-collected samples	No field collected samples were used.
Ethics oversight	All animal experiments were conducted in accordance with the NIH Guide for the Care and Use of Laboratory Animals (National Institutes of Health Publication no. 85-23, revised 1996) and approved by the Institutional Animal Care and Use Committees of City of Hope National Medical Center (Duarte, CA, USA; approval #15023). This statement is provided in the manuscript.

Note that full information on the approval of the study protocol must also be provided in the manuscript.
